# Childhood overeating is associated with adverse cardiometabolic and inflammatory profiles in adolescence

**DOI:** 10.1038/s41598-021-90644-2

**Published:** 2021-06-14

**Authors:** Christopher Hübel, Moritz Herle, Diana L. Santos Ferreira, Mohamed Abdulkadir, Rachel Bryant-Waugh, Ruth J. F. Loos, Cynthia M. Bulik, Deborah A. Lawlor, Nadia Micali

**Affiliations:** 1 Social, Genetic and Developmental Psychiatry Centre, Institute of Psychiatry, Psychology and Neuroscience, King’s College London, London, UK; 2 UK National Institute for Health Research (NIHR) Biomedical Research Centre for Mental Health, South London and Maudsley Hospital, London, UK; 3 National Centre for Register-Based Research, Department of Economics and Business Economics, Aarhus University, Aarhus, Denmark; 4 Department of Medical Epidemiology and Biostatistics, Karolinska Institutet, Stockholm, Sweden; 5 Department of Biostatistics and Health Informatics, Institute of Psychiatry, Psychology and Neuroscience, King’s College London, London, UK; 6 Medical Research Council Integrative Epidemiology Unit at the University of Bristol, Bristol, UK; 7 Population Health Sciences, Bristol Medical School, University of Bristol, Bristol, UK; 8 Department of Pediatrics Gynaecology and Obstetrics, Faculty of Medicine, University of Geneva, Geneva, Switzerland; 9 Department of Psychiatry, Faculty of Medicine, University of Geneva, Geneva, Switzerland; 10 Maudsley Centre for Child and Adolescent Eating Disorders, Michael Rutter Centre for Children and Young People, Maudsley Hospital, London, UK; 11 Icahn School of Medicine At Mount Sinai, New York, NY USA; 12 Department of Psychiatry, University of North Carolina At Chapel Hill, Chapel Hill, NC USA; 13 Department of Nutrition, University of North Carolina At Chapel Hill, Chapel Hill, NC USA; 14 Bristol National Institute of Health Research Biomedical Research Centre, Bristol, UK; 15 Great Ormond Street Institute of Child Health, University College London, London, UK

**Keywords:** Lipidomics, Metabolomics, Predictive markers

## Abstract

Childhood eating behaviour contributes to the rise of obesity and related noncommunicable disease worldwide. However, we lack a deep understanding of biochemical alterations that can arise from aberrant eating behaviour. In this study, we prospectively associate longitudinal trajectories of childhood overeating, undereating, and fussy eating with metabolic markers at age 16 years to explore adolescent metabolic alterations related to specific eating patterns in the first 10 years of life. Data are from the Avon Longitudinal Study of Parents and Children (*n* = 3104). We measure 158 metabolic markers with a high-throughput (^1^H) NMR metabolomics platform. Increasing childhood overeating is prospectively associated with an adverse cardiometabolic profile (i.e., hyperlipidemia, hypercholesterolemia, hyperlipoproteinemia) in adolescence; whereas undereating and fussy eating are associated with lower concentrations of the amino acids glutamine and valine, suggesting a potential lack of micronutrients. Here, we show associations between early behavioural indicators of eating and metabolic markers.

## Introduction

Childhood obesity is a worldwide health problem^1^ and this is particularly concerning as childhood obesity tracks into adulthood^2^. Individuals with larger bodies face discrimination and stigmatisation, which may perpetuate their eating problems and the adverse impact of high BMI on health^3,4^. Developmental programming suggests that early nutrition and lifestyle have long-term effects on later health and the risk of noncommunicable diseases. Persistent obesity throughout childhood is associated with cardiometabolic disease, type 2 diabetes^5^, and cancer^6^, all representing chronic health conditions that severely reduce quality of life and are associated with poor mental health^7^.

The interaction between eating behaviours and our changing environment has played a vital role in the rise of childhood obesity over the last four decades^8^. Combinations of genetic, behavioural, and environmental factors are responsible for weight gain over time^9^. For instance, children living in an obesogenic environment^10^ are exposed to highly palatable, energy dense food while being sedentary most of the time^11^. These environmental conditions may motivate children with an avid appetite^12^ to consume large quantities of energy dense food, which can result in weight gain^13,14^. Findings that children’s eating is prospectively associated with their body mass support the proposed model^15–18^ that eating behaviour is involved in long-term body weight regulation.

Several aetiological factors contribute to childhood obesity, but little is known about underlying biochemical mechanisms that might relate these to later adult diseases. High throughput metabolomics platforms can unveil mechanisms by measuring metabolic profiles that consist of several hundred metabolic markers (e.g., metabolites, lipids, and proteins) in human specimens like plasma. Metabolic profiles represent intermediate phenotypes between eating behaviour and health outcomes. For instance, individuals with obesity have higher lipid concentrations than those at normal weight^19^ and early alterations in metabolic profiles associated with obesity can be observed in childhood^20^. These preliminary findings suggest that metabolic markers are potential biomarkers of noncommunicable disease in children.

Only a few studies have modelled the association between eating behaviour and metabolic markers directly. For instance, metabolic markers have been associated with healthy dietary patterns in postmenopausal women^21^ or the intake of certain food groups in adult English twins^22^, showing specific associations between eating and the human metabolic profile. Eating behaviours, including overeating, undereating, and fussy eating, typically develop in childhood and are influenced by both biological and environmental factors^12,23^. The few studies that have examined metabolomic markers in childhood are either cross-sectional^20^, based on small samples^24,25^, measure only a selected set of markers^25^, or aggregate data from children, adolescents, and adults, introducing extensive heterogeneity and complicating interpretations^26^. Most studies in children and adolescents associate these metabolic profiles with body composition traits, whereas the children’s eating behaviour has not been modeled explicitly.

We have previously provided evidence that parent-reported eating behaviours during the first ten years of life follow longitudinal trajectories^27^ in the large UK population-based Avon Longitudinal Study of Parents and Children (ALSPAC)^28,29^. These trajectories cover three key aspects of eating behaviour: overeating, undereating, and fussy eating, and are associated with body mass index (BMI) at age 11 years^17^ as well as disordered eating at age 16 years^27^. Here, we explore if these childhood eating behaviour trajectories^17^ are associated with metabolic profiles at 16 years assessed by a high-throughput (^1^H) NMR metabolomics platform, using longitudinal data from a subsample of the ALSPAC cohort. Furthermore, we investigate if the relationship between eating behaviour in childhood and metabolic markers in adolescence at 16 years is mediated through BMI measured at 12 years of age. Modelling eating behaviour and body composition jointly to disentangle direct associations of eating with the metabolic profile from indirect associations mediated through BMI represents a unique approach that allows us to differentiate eating-related from body mass-related biomarkers.

## Results

**Figure 1:**
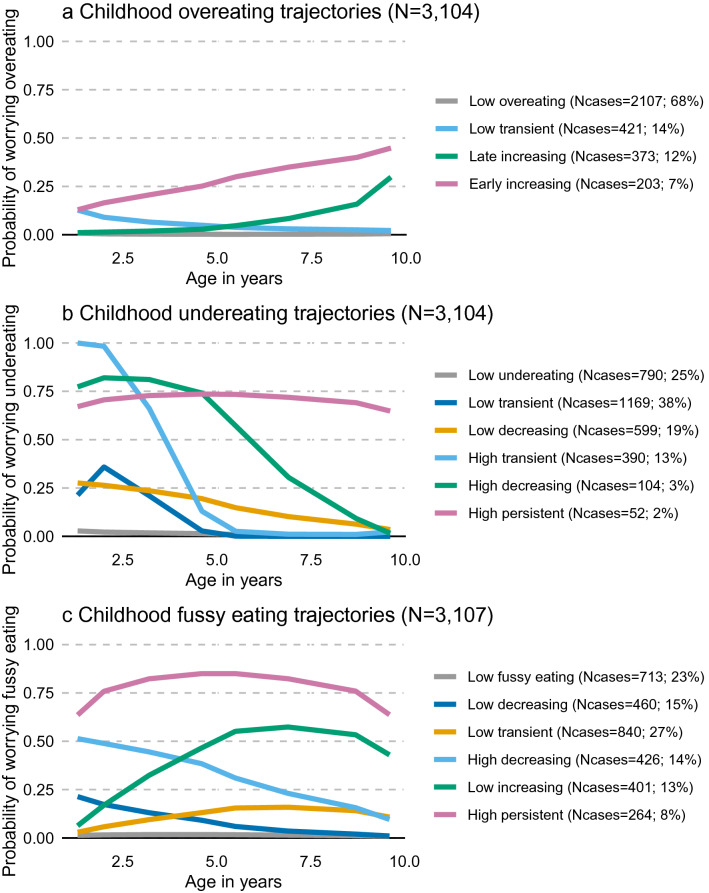
Trajectories of parent-reported child eating behaviours: overeating, undereating, and fussy eating. Trajectories are created using latent class growth analysis. Participants (n = 3107) with at least one measure of eating behaviour and metabolic data at 16 years in the Avon Longitudinal Study of Parents and Children are included.

**Figure 2:**
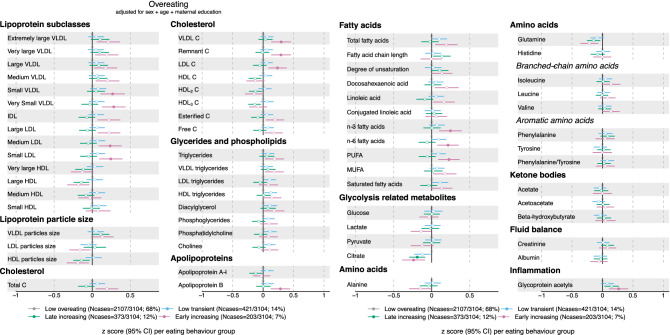
Estimates refer to change in standardised metabolic marker concentration at 16 years in each overeating trajectory, in reference to the “low overeating” trajectory (gray dot). Error bars = 95% confidence intervals (CI). For lipoprotein subclasses, the total lipids (= triglycerides + phospholipids + total cholesterol) point estimate (and CIs) of each 14 subclasses is presented. Estimated beta coefficients and corresponding 95% CIs for particle concentration and specific lipids in each lipoprotein subclass are given in Figure S1a–c, Supplementary Material and Table S1, Supplementary File. Analyses adjusted for sex, age at metabolite measure and maternal education. Note: Association where CIs do not cross 0, p-value < 0.05; Filled dot: Association meets the p-value threshold of < 0.003. *C* cholesterol, *IDL* intermediate-density lipoprotein, *LDL* low-density lipoprotein, *HDL* high-density lipoprotein, *VLDL* very low-density lipoprotein, *MUFA* monounsaturated fatty acids, *PUFA* polyunsaturated fatty acids. *Note* Filled dot: CI do not include the null after multiple testing correction. MUFA, PUFA and saturated fatty acid concentrations include all fatty acids detected which have one, more than one, or zero C=C double bonds in their backbone, respectively.

**Figure 3:**
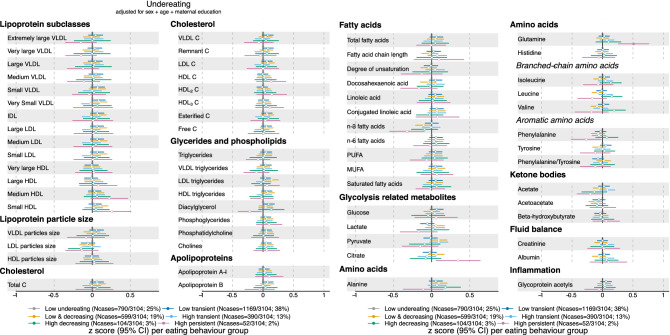
Estimates refer to change in standardised metabolic trait concentration at 16 years in each undereating trajectory, in reference to the “low undereating” trajectory (gray dot). Error bars = 95% confidence intervals (CI). For lipoprotein subclasses, the total lipids (= triglycerides + phospholipids + total cholesterol) point estimate (and CIs) of each 14 subclasses is presented. Estimated beta coefficients and corresponding 95% CIs for particle concentration and specific lipids in each lipoprotein subclass are given in Figure S2a–c, Supplementary Material and Table S2, Supplementary File. Analyses adjusted for sex, age at metabolite measure and maternal education. *Note* Association where CIs do not cross 0, p-value < 0.05; Filled dot: Association meets the p-value threshold of < 0.003. *C* cholesterol, *IDL* intermediate-density lipoprotein, *LDL* low-density lipoprotein, *HDL* high-density lipoprotein, *VLDL* very low-density lipoprotein, *MUFA* monounsaturated fatty acids, *PUFA* polyunsaturated fatty acids. *Note* Filled dot: CI do not include the null after multiple testing correction. MUFA, PUFA and saturated fatty acid concentrations include all fatty acids detected which have one, more than one, or zero C=C double bonds in their backbone, respectively.

**Figure 4:**
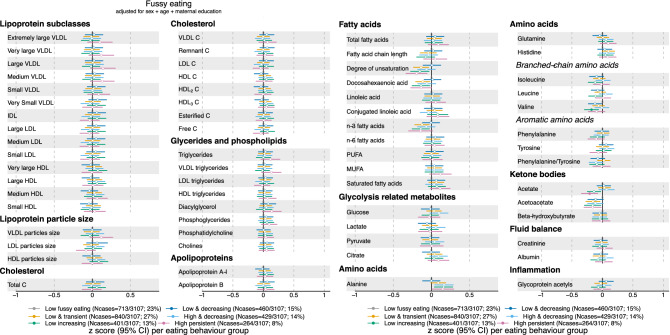
Estimates refer to change in standardised metabolic trait concentration at 16 years in each fussy eating trajectory, in reference to the “low fussy eating” trajectory (gray dot). Error bars = 95% confidence intervals (CI). For lipoprotein subclasses, the total lipids (= triglycerides + phospholipids + total cholesterol) point estimate (and CIs) of each 14 subclasses is presented. Estimated beta coefficients and corresponding 95% CIs for particle concentration and specific lipids in each lipoprotein subclass are given in Figure S3a–c, Supplementary Material and Table S3, Supplementary File. Analyses adjusted for sex, age at metabolite measure and maternal education. *Note* Association where CIs do not cross 0, p-value < 0.05; Filled dot: Association meets the p-value threshold of < 0.003. *C* cholesterol, *IDL* intermediate-density lipoprotein, *LDL* low-density lipoprotein, *HDL* high-density lipoprotein, *VLDL* very low-density lipoprotein, *MUFA* monounsaturated fatty acids, *PUFA* polyunsaturated fatty acids. *Note* Filled dot: CI do not include the null. MUFA, PUFA and saturated fatty acid concentrations include all fatty acids detected which have one, more than one, or zero C=C double bonds in their backbone, respectively.

**Figure 5:**
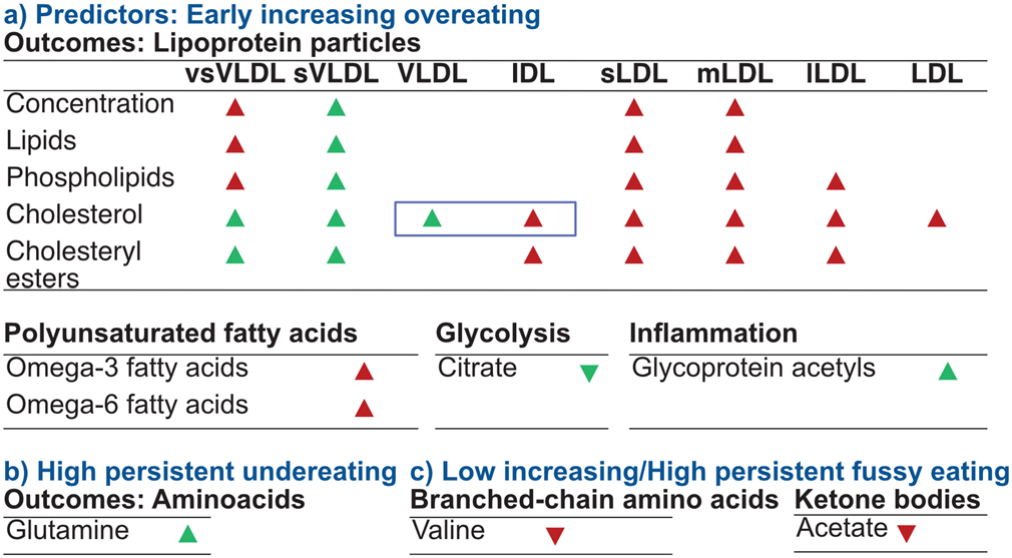
Overview of significant associations between eating trajectories and metabolic markers and their mediation through body mass index (BMI) at age 12 years in the Avon Longitudinal Study of Parents and Children (ALSPAC, n = 3104). Arrows in upward direction indicate that the metabolic marker is measured to be higher in children with either high and persistent overeating, undereating, or fussy eating compared with children that are not reported by their parents to engage in any of these eating behaviours. A red arrow indicates that the eating behaviour is associated with the higher metabolite independent from the adolescents’ body mass index (BMI) at age 12 years compared to the reference category. Green arrows indicate that the association is mediated through BMI at age 12 years. The blue square indicates remnant cholesterol.

In 3104 adolescents, a subsample of the original ~ 14,000 ALSPAC cohort (Table 1), we test the prospective association between parent-reported eating behaviour in the first ten years of life (Fig. 1a–c) and metabolic profiles at the age of 16 years. Trajectories of overeating (Fig. 2), undereating (Fig. 3) and fussy eating (Fig. 4) in childhood differ in their metabolic profile at age 16 years (Fig. 5).

**Table 1:** Eating behaviours and characteristics of the subsamples of the Avon Longitudinal Study of Parents and Children (ALSPAC) that were included in the construction of the eating behaviour trajectories using latent class growth analysis^27^ and the subsample that also has an available metabolic profile at age 16 years.

Characteristics	Participants at baseline (alive at one year and part of the original core ALSPAC cohort (*n* = 13,783)	Participants with at least one measure of eating behaviour during their first 10 years (*n* = 12,048)	Participants with at least one measure of eating behaviour and metabolic markers at 16 years (*n* = 3107)	
		Mean (SD) or *n* (%)	Mean (SD) or *n* (%)	
**Sex**				
Boys	7111 (52%)	6208 (52%)	1499 (48%)	
Girls	6672 (48%)	5840 (48%)	1608 (52%)	
**Maternal education**				
Less than A-levels	7917 (65%)	7271 (60%)	1558 (52%)	
A-levels or higher	4330 (35%)	4831 (40%)	1459 (48%)	
BMI at 12 years (kg/m^2^)	19.1 (3.4), n = 6651	19.1 (3.4), n = 6548	19.1 (3.3), n = 2832	
BMI at 16 years (kg/m^2^)	21.5 (3.6), n = 5086	21.5 (3.6), n = 4993	21.5 (2.4), n = 3067	
**Eating behaviour trajectories**				Mean BMI at 16 years per EB trajectory in analyses sample
**Overeating**				
Low overeating		8240 (69%)	2107 (68%)	21 (3.1)
Low transient		1756 (15%)	421 (14%)	21.6 (3.2)
Late increasing		1276 (11%)	373 (12%)	23.3 (3.9)
Early increasing		730 (6%)	203 (6%)	23.8 (4.5)
**Undereating**				
Low undereating		2940 (24%)	790 (25%)	21.8 (3.5)
Low transient		4413 (37%)	1169 (38%)	21.6 (3.6)
Low and decreasing		2454 (20%)	599 (19%)	21.4 (3.4)
High transient		1548 (13%)	390 (13%)	21.1 (3.5)
High decreasing		437 (4%)	104 (3%)	21.2 (3.2)
High persistent		214 (2%)	52 (2%)	19.5 (2.7)
**Fussy eating**				
Low fussy eating		2713 (23%)	713 (23%)	21.9 (3.6)
Low decreasing		1718 (14%)	460 (15%)	21.9 (3.6)
Low transient		3272 (27%)	840 (27%)	21.5 (3.4)
High and decreasing		1710 (14%)	429 (14%)	21.2 (3.5)
Low increasing		1590 (13%)	401 (13%)	21.3 (3.4)
High persistent		1045 (9%)	264 (8%)	20.6 (3.3)

*BMI* body mass index, *EB* eating behaviour, *n* number, *SD* standard deviation.

We illustrate the results in forest plots, presenting standardised β coefficients with corresponding 95% confidence intervals (95% CI). Unstandardised estimates are reported in Supplementary Tables S1–S3. Each eating behaviour trajectory is compared with the “low stable” reference trajectory, see grey colour in Figs. 1, 2 and 3 and in Supplementary Figs. S1–S3. Positive estimates (i.e., to the right of the black reference line) indicate that this metabolic marker is higher in adolescents that were assigned to the corresponding eating behaviour trajectory in childhood compared with the reference “low stable” trajectory. Negative estimates (i.e., to the left of the black reference line) indicate the opposite. Filled-in dots in the plot represent associations that were statistically significant with a *p* value lower than the significance threshold of 0.003 after multiple testing correction. We grouped the metabolic markers by their chemical structures (e.g., fatty acids) or metabolic function (e.g., glycolysis). Information on lipoprotein subclasses is presented in Supplementary Methods and Supplementary Figs. S1–S3.

We present results without BMI adjustment as primary results, because we assume that BMI lies on the causal pathway between eating behaviour and metabolic markers. Alternatively, higher BMI may be a result of the changes in metabolic markers and conditioning on BMI would introduce a collider in this case. However, sensitivity analyses adjusted for BMI can be found in Supplementary Tables S1–S3.

Moreover, to systematically investigate the potential mediating effect of BMI on the association between eating behaviour and metabolic profile, we perform mediation analysis using structural equation modelling. See Supplementary Fig. 4 for an illustration of the mediation model. The eating behaviours trajectories are the exposure, BMI at age 12 years is the mediator and the metabolic profile at age 16 years is the outcome. A total of 37 significant associations, discovered in the main analyses, were carried forward for the mediation analyses. Absence of mediation is indicated by a non-significant indirect association but significant direct association, for example, there is no significant indirect association via BMI at 12 years for the association between increasing overeating and the omega-3 fatty acid concentration. A significant indirect path implies mediation via intermediate BMI at age 12 years.

### Adolescent metabolic profile of children who overeat in childhood

Children that are reported to be overeating during the first ten years of life show the following metabolic profile at age 16 years.

#### Very low-density lipoprotein (VLDL) particles

Children who reportedly overate early in life and with an increasing pattern of overeating during the first ten years of life (Fig. 1a, pink line) have a 0.23 standard deviation (SD; 95% CI 0.08, 0.38; *p* = 0.002) higher concentration of very small VLDL particles than children who are not reported to overeat. This association is not mediated through the children’s BMI at age 12 years. Additionally, these children have a 0.24 SD (95% CI 0.08, 0.39; *p* = 0.002) higher concentration of small VLDL than children who do not overeat and the association is mediated through the children’s BMI at age 12 years.

The very small and small VLDL particles of children with early increasing overeating contain more lipids and phospholipids than those of children who do not overeat (Supplementary Fig. 1). Similar to the total particle concentration, the higher concentrations of lipids and phospholipids within the small VLDL particles is associated with childhood overeating mediated through BMI at age 12 years but not in very small VLDL particles. Detailed results for the lipoprotein content (i.e., subclasses) are presented in Supplementary Tables S1-3 and Supplementary Figs. 1–3.

Additionally, cholesterol and cholesteryl esters are present in higher concentrations in very small VLDL particles of children who reportedly started overeating early and do so increasingly over the first ten years of life compared with children that do not overeat (Supplementary Table S1). In contrast to the lipid concentrations in very small VLDL particles, the association between higher concentrations of cholesterol and cholesteryl esters in very small VLDL and early increasing overeating is mediated through the children’s BMI at age 12 years (Supplementary Table S4).

#### Intermediate-density lipoprotein (IDL)

Independent of their BMI at age 12 years, children with reported early increasing overeating have higher concentrations of cholesterol and cholesteryl esters in their IDL particles at age 16 years than children that do not overeat (Supplementary Table S1).

#### Remnant cholesterol

Children with early increasing overeating show a 0.29 SD (95% CI 0.14, 0.45; *p* = 2.4 × 10^–4^) higher concentration of remnant cholesterol at 16 years than children that do not overeat. This association is mediated through BMI at age 12 years (Supplementary Table S4). Remnant cholesterol summarises the total cholesterol contained in IDL and VLDL particles.

#### Low-density lipoprotein (LDL)

Children with early and increasing overeating patterns have a 0.24 SD (95% CI 0.09, 0.39; *p* = 0.002) higher concentration of small and medium LDL particles than children who do not overeat. These small and medium LDL particles contain higher concentrations of lipids, phospholipids, cholesterol, and cholesteryl esters than those LDL particles of children who do not overeat (detailed results, see Supplementary Table S1).

Additionally, children with early increasing overeating have higher concentrations of phospholipids, cholesterol, and cholesterol esters in their large LDL particles than children who do not overeat. These higher concentrations and altered compositions of small, medium, and large LDL particles are directly associated with the overeating that occurred in childhood independent of the children’s BMI at age 12 years (Supplementary Table S4).

#### Apolipoprotein B

Children reported to early and increasingly overeat during the first ten years of life have a 0.28 SD (95% CI 0.13, 0.44; *p* = 3.4 × 10^–4^) higher concentration of apolipoprotein B in adolescence than children who do not overeat. This association is mediated through the children’s BMI at age 12 years.

#### Polyunsaturated fatty acids (PUFA)

Besides these alterations in lipoprotein particles, children that early and increasingly overeat also have higher concentrations of polyunsaturated fatty acids, including omega-3 (β = 0.25 SD, 95% CI 0.10, 0.39; *p* = 9.5 × 10^–4^) and omega-6 fatty acids (β = 0.21 SD, 95% CI 0.08, 0.35; *p* = 0.002) than children who do not overeat, independent from their BMI at age 12 years. This is expected because the human body does neither produce omega-3 nor omega-6 fatty acids itself. Providing evidence for the additional value of modelling eating behaviour independent from measures of body mass (e.g., BMI or body fat percentage).

#### Glycoprotein acetyls

Additionally, children who overeat early and increasingly during the first ten years of life have a 0.27 SD (95% CI 0.11, 0.42; *p* = 7.1  × 10^–4^) higher concentration of glycoprotein acetyls, markers of chronic inflammation. This association is mediated through their BMI at age 12 years.

#### Citrate

Children who are reported to overeat early and increasingly (β = − 0.24 SD, 95% CI − 0.39, − 0.09; *p* = 0.002) or late and increasingly (β = − 0.19 SD, 95% CI − 0.29, − 0.09; *p* = 2.7 × 10^–4^) have lower concentrations of citrate than children who do not. The association between increasing overeating and lower citrate concentration is mediated through BMI at 12 years.

The results for undereating and fussy eating are described below. For a list of all results, see Supplementary Tables S1–S3 and a full list of all mediation models is in Supplementary Table S4.

### Adolescent metabolic profile of children who undereat in childhood

Children with reported high and persistent undereating (Fig. 1b, pink line) have a 0.52 SD (95% CI 0.26, 0.80; *p* = 6.4 × 10^–5^) higher concentration of glutamine at age 16 years than children that do not undereat (Fig. 3). Some mediation is suggested by a significant indirect association (β = 0.05, 95% CI 0.02, 0.08), indicating that a small portion of the association is mediated via BMI at 12 years, whereas the majority of the association is captured by the direct association (β = 0.48, 95% CI 0.20, 0.76).

### Adolescent metabolic profile of children who show fussy eating in childhood

Children reported to show high persistent fussy eating (Fig. 1c, pink line) throughout childhood have a 0.18 SD (95% CI − 0.29, − 0.06; *p* = 0.002) lower concentration of the essential branched-chain amino acid valine, and children showing first low and then increasing fussy eating have a 0.21 SD (95% CI − 0.34, − 0.08; *p* = 0.002) lower acetate concentration than children who do not show fussy eating (Fig. 4). Both associations between fussy eating and the metabolic marker are not mediated by BMI at age 12 years.

## Discussion

In this comprehensive study, we investigate the association of trajectories of eating behaviours over the first 10 years of life with variation in metabolic markers at age 16. Adolescents who are reported to overeat, undereat, or be fussy about their eating in childhood show alterations in key metabolic markers at the age of 16 years. Adolescents who overeat in childhood have a metabolic profile characterised by chronic inflammation and higher lipid concentrations (i.e., hyperlipidaemia). This metabolic profile is a well-characterised risk factor for a variety of adverse cardiovascular health outcomes, including heart attack and stroke^30,31^. Furthermore, children reported to undereat persistently in childhood have higher concentrations of glutamine in adolescence. Glutamine is an amino acid that serves as an alternative substrate for energy generation if a diet is lacking carbohydrates or other energy sources. The metabolism of a child that undereats might resort to glutamine as an alternative energy source^32^. Lastly, children who are reported to be persistently fussy about their eating have lower concentrations of the essential branched-chain amino acid valine that only can be obtained from the diet^33^. This may indicate a lack of this essential amino acid potentially due to the limited range of food eaten. However, these hypotheses require further investigation including detailed dietary reviews and cannot be directly answered with our observational epidemiological design in the current data set.

Comparison of our results with previous literature is complicated as only few studies used metabolomics platforms to assess metabolic profiles longitudinally. In addition, results from our mediation analyses suggest a mechanism by which eating behaviour patterns impact BMI, which is then reflected in changes in the metabolic profile in adolescence. However this is only true for some metabolomic markers, as some direct associations from eating behaviours to metabolomic markers are indicated to be independent of intermediate BMI.

### Overeating

#### Lipoprotein particles, remnant cholesterol, and apolipoprotein B

We show that children who have been rated by their parents to overeat in an increasing pattern across the first ten years of life have higher concentrations of VLDL, remnant cholesterol, and apolipoprotein B at 16 years of life than children who do not overeat. They, furthermore, have VLDL, IDL, and LDL particles that are altered in their composition carrying more lipids, phospholipids, cholesterol, and cholesteryl esters than those particles of children who are rated by their parents to not overeat. This is of clinical relevance, as these metabolic factors have been associated with adverse health outcomes, such as higher risk for cardiovascular disease^34^.

*VLDL particles* are triglyceride-rich lipoproteins that are created in the liver transporting lipids to the adipose tissue for storage and the muscles for energy production^35^. Pharmaceutical lowering of small VLDL is associated with fewer atherosclerotic cardiovascular disease events in preventative trials^36^.

*Remnant cholesterol* is a summary measure of the cholesterol content of all triglyceride-rich lipoproteins (i.e., VLDL and IDL)^37^. Remnant cholesterol is robustly associated with adverse cardiovascular health outcomes, including atherosclerosis, myocardial infarction^30,31^, and stroke^38^. Remnant cholesterol, moreover, shows an association with premature myocardial infarction before the age of 40 years^39^ especially important in the context of our findings in the developmental ALSPAC cohort. Our results, therefore, propose remnant cholesterol as a potential biomarker for early onset cardiovascular complications.

*Apolipoprotein B* is the primary organisational protein of lipoproteins and binds to the LDL receptor on various cells. Higher apolipoprotein B more accurately indicates risk for cardiovascular disease^40,41^ than other lipoprotein markers^42,43^ because each lipoprotein particle carries exactly one apolipoprotein B particle and based on this number the exact concentration of lipoproteins can be estimated. Apolipoprotein B is a better predictor of cardiovascular diseases than, for instance, LDL cholesterol^42,43^.

#### Glycoprotein acetyls

Additionally, children who began to overeat early in childhood and did so increasingly over the first ten years of life have higher concentrations of glycoprotein acetyls, which indicates a chronic inflammatory state as early as at the age of 16 years. *Glycoprotein acetyls* are heterogeneous nuclear magnetic resonance signals summarising five circulating glycoproteins that are primarily produced in the liver^44^. The concentration of glycoprotein acetyls has previously been associated with BMI^45^ paralleling our finding that the association between overeating and glycoprotein acetyls is mediated through BMI at age 12 years. Glycoprotein acetyls are associated with a variety of adverse health outcomes, including inflammation, cardiovascular disease^46^, type 2 diabetes^47^, chronic renal failure, chronic obstructive pulmonary disease, hypertension, inflammatory polyarthritis^48^, and all-cause mortality^49^.

The combination of hyperlipidaemia with higher chronic inflammatory markers represents an adverse cardiometabolic profile that is associated with a variety of noncommunicable diseases in epidemiological studies^34,49–51^. Our study indicates that adolescents who overate in childhood may already have entered this metabolic state or a precursor to it at the age of 16 years.

#### Polyunsaturated fatty acids

Comparing adolescents that overate early and increasingly during childhood with those who did not shows that not only lipids that can be synthesised by the human metabolism, but also lipids that are solely obtained from the diet are elevated^52^. Overeating in childhood was independent of BMI at age 12 associated with essential polyunsaturated fatty acids, including omega-3 (e.g., mainly contained in plant sources and fish oil) and omega-6 (e.g., mainly contained in plant oils) polyunsaturated fatty acids^53^. These long chain polyunsaturated fatty acids are precursors for locally acting bioactive metabolites^54,55^, including eicosanoids like prostaglandins, prostacyclin, and thromboxane^55^, but only the precise action of a few of those metabolites is fully understood^53,56^. Imbalances in these pathways can lead to conditions including thrombosis, inflammation, asthma, and inflammatory bowel disease^57^. Overall, the association among polyunsaturated fatty acids and cardiovascular health^58–60^, diabetes, depression, and neuronal development are controversial and need further in-depth investigation^53^ as prospective studies and meta-analysis show mixed results^58,61–63^. Imbalances in the system are measured by the omega-6/omega-3 ratio. A high omega-6 to omega-3 ratio is associated with obesity and a pro-inflammatory state^64^ as omega-6 fatty acids are often precursors for pro-inflammatory metabolites. The absorption of omega-3 and omega-6 fatty acids is highly dependent on the overall fat content of a meal^65^ that may suggest a higher absorption if a general tendency to overeat exists. However, studies investigating the absorption are extremely small, sampling generally fewer than 20 individuals. The omega-6/omega-3 ratio of adolescents with increasing overeating in childhood in our sample is 10:1, suggesting an overconsumption of omega-6 fatty acids within an unhealthy range.

#### Citrate

Adolescents who were rated to overeat early or later in childhood have lower concentrations of citrate at age 16 years than children who do not overeat. The human blood citrate household is tightly regulated by bone resorption and renal clearance of citrate via the hormones parathyroid hormone, vitamin D, and calcitonin^66^. In cells, citrate is produced in the citric acid cycle in mitochondria^67^ and citrate regulates energy production by inhibiting and inducing strategic enzymes of glycolysis, citric acid cycle, gluconeogenesis, and fatty acids synthesis^68^. Health conditions, such as obesity, are associated with a dysregulation of the mitochondrial oxidation chain^69^ and lower citrate synthase activity in the Krebs cycle^70^, resulting in lower productions of citrate. This dysregulation can be observed in children 8–12 years old^71^ and may reflect the observation that overeating in childhood is associated with lower citrate in adolescence in our study. This association is mediated through BMI at age 12 years potentially indicating a dysfunction of mitochondria at the age of 16 years.

### Undereating

#### Glutamine

Regarding other eating behaviours that we investigate, adolescents who are rated to undereat persistently during childhood show higher glutamine concentrations in adolescence than those who do not. Glutamine is the most abundant amino acid in the human body, a basic building block for proteins, and a viable energy source^72^. Glutamine can become conditionally essential under stress^32^. As a biomarker, higher plasma glutamine is associated with lower BMI accompanied by lower triglycerides, lower insulin, and higher high-density lipoportein concentrations^73^, which overall represents a favourable cardiometabolic profile^74^. Cross-sectionally and longitudinally, higher glutamine is associated with lower risk of developing type 2 diabetes^75,76^ and oral administration of glutamine improves glycemic traits in small pilot studies^77,78^. In summary, this suggests glutamine as a biomarker for a favourable cardiometabolic profile that is associated with parent-reported undereating in childhood in our sample. Validation of this association is needed.

### Fussy eating

#### Valine

Adolescents with first low then rapidly increasing fussy eating in childhood have lower concentrations of the essential amino acid valine in adolescence than those without fussy eating. Valine is one of three essential branched-chain amino acids (i.e., leucine, isoleucine, and valine) that are not synthesised by the human body^79^. Branched-chain amino acids regulate several pivotal metabolic pathways^33,80^, are elevated in prediabetes^81,82^, and positively associated with insulin resistance and overweight in adults^83–85^ and children^24,25,86^. Regarding the association between branched-chain amino acids and insulin resistance, cause and effect are not yet fully understood, but an branched-chain amino acid-related uncoupling of intracellular insulin signalling has been proposed^33^. The cross-sectional character of two of the studies in youth^24,86^ and the extremely small sample (*n* = 17) of the longitudinal investigation limit the interpretability of the results severely^25^. As most studies investigate individuals above the age of 18 years^84^, here, we extend the findings to adolescents. Our findings suggest that children who are deemed by their parents to be persistent fussy eaters in childhood may benefit from branched-chain amino acid supplements as they seem to lack essential amino acids in adolescence.

#### Acetate

Adolescents who are high persistently fussy about their eating in childhood have lower concentrations of the short-chain fatty acid acetate than those who are not fussy. One of the main products of colonic microbial fermentation of unabsorbed dietary fibre are short-chain fatty acids (e.g., acetate, propionate, and butyrate)^87^. However, short-chain fatty acids are also endogenously produced as a result of elevated fat oxidation^88,89^. About 44% of whole-body acetate production is estimated to be gut microbiota derived^90^ and several mechanisms are forwarded describing the involvement of acetate in the regulation of appetite and energy homeostasis. For instance, intracellular receptors for short-chain fatty acids are expressed in gut epithelium and adipose tissue^91^, but short-chain fatty acids also seem to have direct central nervous appetite regulating effects^92^. Higher systemic acetate concentrations suppress food intake^93^, reduce lipolysis and plasma free fatty-acid concentrations and thus improve insulin sensitivity^94^. Acetate is also negatively associated with visceral adipose tissue^95^ and oral acetate reduces the number of free fatty acids in human blood^96^ while rectal infusion of acetate increases serum cholesterol, glucagon, and acetate concentrations and reduces free fatty acids within 30 min^97^. In the light of this evidence, and a potential BMI mediated association in our study, individuals that are reported to be fussy about eating in childhood may have altered microbiota leading to a lower colonic acetate production and absorption.

### Limitations

We present our results in the light of the following limitations. First, dietary habits and eating behaviour may be better reflected in the urinary metabolome. Presumably due to high metabolite turn-over, metabolites in urine are more closely associated with diet than those in serum^98,99^. Second, our subsample of the ALSPAC cohort may be selected as it differs with regards to maternal education and variability in BMI at age 16 years from the core cohort (Table 1) limiting generalisability. Third, due to the overall available sample size, some derived eating behaviour trajectories included only a few individuals (e.g., *n* = 53) reducing power and also limiting the ability to examine sex differences^100,101^. We, therefore, recommend replication and extension of our findings including investigations of sex differences in larger cohorts as soon as a large childhood cohort that assesses metabolomic profiles and eating behaviour prospectively becomes available. Fourth, it is not yet possible to measure the entire metabolome with a single or a combination of metabolomics technologies^102^. Nonetheless, our study characterised one of the broadest molecular signatures of systemic metabolism available at date of analysis. Fifth, plasma samples were analysed by NMR after 7 years of storage at − 80 °C. Previous work has suggested that storage longer than 5 years can result in altered levels of lipids, amino acids, and hexoses^103^. However, more recently, a study combining metabolite data from different cohorts with varying storage times indicated that even though older samples show some degradation of concentration, this does not impact standard deviation-scaled measures as used in our epidemiological analyses^104^. Sixth, given our data set for secondary data analysis, we were not able to investigate potential batch effects during the metabolic marker measurement directly. However, studies combining several cohorts using the same NMR platform showed acceptable coefficients of variation^105,106^. Seventh, the childhood eating behaviour is reported by parents. However, in a population cohort sampling ~ 15,000 children, this is the only viable method to obtain information on eating behaviour during the first ten years of life. Eighth, our investigation did not include measures of genetic liability for metabolic alterations. Future investigations should include, for instance, polygenic scores to model polygenic liability for metabolic alterations^107^. Finally, our paper focuses on eating behaviours and did not analyse information on diet quality or dietary intake, and further investigations including these measures in addition to eating behaviours is warranted.

## Conclusion

Our findings lend further evidence to the merit of modelling eating behaviour as differential longitudinal trajectories capturing variance and identifying subgroups of eating behaviour throughout the first ten years of life as we were able to associate these trajectories with meaningful biochemical markers, including lipid, lipoprotein, glycolytic, amino acids and an inflammatory marker. This study represents an important first step in understanding how childhood eating behaviours can impact blood biomarkers in later life. Further replication in independent samples is needed, as well as more research on the health impact of metabolic profile in adolescence on health in adulthood. If future research validates these initial findings, we could speculate that metabolic biomarkers in combination with parent-reported behaviours^108^ may represent a potential approach to target children at risk for preventative intervention aiming to hopefully curb the development of cardiometabolic illness later in life^109^.

## Methods

### Sample

Participants included in this study are a subsample of adolescents of the population-based ALSPAC cohort (*n* = 3107) that sampled mothers and their children born in the southwest of England^28,29,110^. All pregnant women that were expected to have a child in the time period between 1 April 1991 and 31 December 1992 were contacted to participate in the original cohort. At the beginning, 14,451 pregnant women took part and 13,988 children were alive at the end of year one. To guarantee independence of individuals, one sibling per set of multiple births (*n* = 203 sets) is randomly included in our analysis. The study website contains details of all the data that is available through a fully searchable data dictionary and variable search tool (www.bristol.ac.uk/alspac/researchers/our-data/).

### Ethics approval and consent to participate

Ethical approval for the ALSPAC participants was obtained from the ALSPAC Ethics and Law Committee and the Local Research Ethics Committees: www.bristol.ac.uk/alspac/researchers/research-ethics/. The main caregiver initially provided consent for child participation, and from the age 16 years, the offspring themselves have provided informed written consent. At 16 years old, sole consent from the study child was considered acceptable by the Committee and although the law was not specific about young people with regards to research, this complied with the Family Law Reform Act 19695 as regards treatment: those who are 16 years old or above have the same legal ability to consent to any medical, surgical or dental treatment as anyone above 18, without consent from their parent or guardian^111^. Children were invited to give assent where appropriate. Consent for biological samples was collected in accordance with the Human Tissue Act (2004) and informed consent for the use of data collected via questionnaires and clinics was obtained from participants following the recommendations of the ALSPAC Ethics and Law Committee at the time. All methods were carried out in accordance with relevant guidelines and regulations.

### Eating behaviour trajectories during childhood

We obtain longitudinal trajectories of overeating, undereating, and fussy eating based on repeatedly parent-reported eating behaviours during childhood. Parents report their child’s eating behaviour for the past year at the ages of 1.3, 2.0, 3.2, 4.6, 5.5, 6.9, 8.7 and 9.6 years^27^. Using latent class growth analyses^112,113^, we derive trajectories including children that have data on at least one time point (*n* = 12,002), using full information maximum likelihood under the missing at random (MAR) assumption. We obtain four trajectories of overeating, six of undereating, and six of fussy eating (Fig. 1).

### Sample with available metabolic profile

This analysis includes a subsample of the data presented in Herle et al.^27^ as it includes adolescents with parent-reported childhood eating behaviour, metabolic profile at age 16 years, and available covariates: sex, age at metabolite measurement, and maternal education at birth (*n* = 3107). The sample has an average age of 15.5 years (SD = 0.4), and average BMI of 21.5 kg/m^2^ (SD = 2.4), and includes 1608 (52%) girls. The number of individuals in each eating behaviour trajectory is presented in Table 1. Our subsample has a similar sex distribution as the original core ALSPAC cohort, but differs with regards to maternal education and variability in BMI at age 16 years (for details, see Table 1).

### Metabolic profile at age 16

In adolescents’ fasted EDTA plasma, we measure 158 metabolic markers using the high-throughput targeted ^1^H nuclear magnetic resonance (NMR) metabolomics platform, Nightingale Health (Helsinki, Finland). The platform contains one single experimental setup and is located at the University of Bristol. The read out includes routine lipids, 14 lipoprotein subclasses with their particle concentration and their contained lipids, see Supplementary Methods. Additionally, fatty acids, their chain length, degree of unsaturation and others are measured, see Supplementary Methods (i.e., lipoprotein subclasses and fatty acids details). Moreover, we measure amino acids, glycolysis-related, and gluconeogenesis-related metabolites and their metabolites: ketone bodies. We additionally assess creatinine, and an inflammation-related metabolic marker. These metabolic markers are a broad molecular signature of the human metabolism^114^ and are assessed in clinically meaningful concentrations (e.g., glucose concentration in mmol/L). We present fatty acids in original units as well as proportions (%) of total fatty acids. In addition, we calculated the phenylalanine/tyrosine ratio as it is a marker of catabolism^115,116^.

### Nuclear magnetic resonance (NMR) spectroscopy methods

#### Processing of samples

Plasma samples were collected when participants were on average 16 years old. All samples were processed and transferred for storage at − 80 °C within 4 h. The samples were stored for seven years before processing. The stability of the samples was guaranteed through surveillance of the freezing temperature. NMR-based metabolomics was measured in 2013 from stored blood samples for adolescents collected at the 2006 follow-ups in the clinics.

Prior to sample analysis, frozen samples were thawed in the refrigerator (4 °C) overnight, prepared with a sodium phosphate buffer, randomized and then immediately run through the NMR spectrometer. Aliquots of each sample were transferred into 3-mm outer-diameter NMR tubes and mixed in 1:1 ratio with a phosphate buffer (75 mM Na_2_HPO_4_ in 80%/20% H_2_O/D_2_O, pH 7.4, including also 0.08% sodium 3-(trimethylsilyl) propionate-2,2,3,3-d_4_ and 0.04% sodium azide) automatically with an automated liquid handler (PerkinElmer Janus Automated Workstation). Each rack of 96 samples takes about 9 h 22 min to run and contains two control samples to check for any degradation. These are (1) a technical control made from glucose and alanine, as well as (2) a serum control made from pooled serum. The control samples are used to track the stability of the spectrometer over time, independent of plasma and serum samples from the given cohort or other cohorts. Batch effects are negligible as Nightingale Health is a targeted platform.

#### Metabolomics quantification

Using a Bruker AVANCE III spectrometer operating at 500 or 600 MHz that measures through three molecular windows in each sample, the metabolites are quantified. From serum with added phosphate buffer, two of the spectra (LIPO and LMWM windows) at 37 °C, and from serum lipid extracts at 22 °C, one spectrum (LIPID window), that requires minimal preparation, are acquired. Details of the platform and its use in epidemiological studies are described in detail^117–122^. Several genetic and observational epidemiological studies use the same platform^123–125^. Supplementary Table S5 lists the quantified metabolites including their units.

#### Quality control

In an automated procedure, we analyse NMR spectra to quantify absolute molar metabolite concentrations. Prior to analyses these pre-processing steps were undertaken: Fourier transformations to NMR spectra, automated phase correction, overall signal check for missing/extra peaks, background control, baseline removal, and spectral area-specific signal alignments^122^. We quantify each metabolite via a ridge regression model to adjust for heavily overlapping spectra. We quantify lipoproteins and lipids through calibrating against high performance liquid chromatography, and individual cross-validation against NMR-independent lipid data. We quantify low-molecular-weight metabolites and lipid extract measures (mmol/l) applying regressions calibrated against several manually fitted metabolite measures. We quantify our calibration data using an iterative line-shape fitting analysis (PERCH NMR software, PERCH Solutions Ltd., Kuopio, Finland).

As lipid extraction protocols show experimental variation, we cannot directly quantify lipid extract measures absolutely. Thus, we used the total cholesterol quantified from the native serum LIPO spectrum to scale the serum extract metabolites. Measures from our platform are strongly correlated and highly agree with routine clinical chemistry measurements of total cholesterol, low density lipoprotein cholesterol, high-density lipoprotein cholesterol, triglycerides and glucose assessed on the same plasma/serum samples^117,122^. For details of the LIPO window spectrum, see the Supplementary Methods (i.e., LIPO window).

### Statistical analyses

Prior to analyses, metabolites with a negative value were assigned as missing as negative concentrations are not possible, and distributions of metabolite measures were screened for implausible maximum values. For comparison purposes across metabolic markers and to facilitate plotting, we standardise all metabolic markers (i.e., subtraction of the mean and subsequent division by the standard deviation [SD]). We fit linear regressions associating the childhood eating behaviour trajectories as categorical independent variables with the metabolic markers at 16 years as outcomes. We use the normative trajectory “low stable” (i.e., grey color in plots) representing no overeating, undereating, or fussy eating as a reference category. This reference category includes children that are rated as not overeating, not undereating, or not being fussy about eating during the first 10 years of life.

Our main analysis includes sex, age at metabolite measurement, and maternal education at birth (considered as relevant for eating behaviour during the first ten years of life) as confounders. As a sensitivity analysis, to investigate the potential influence of BMI on the associations, we adjust the regression models for BMI at age 16 years.

In order to correct for multiple testing, we adjust the *p* value threshold, using the Bonferroni method. However, the metabolites are highly intercorrelated; therefore, we perform a principal component analysis on the correlation matrix of all measured metabolic markers, and identify 20 components explaining the most variance. We use this number of principal components to adjust the *p* value threshold. This results in a Bonferroni-adjusted *p* value threshold of 0.003. All analyses are conducted using Stata version 16.1 and R version 3.5.1.

#### Mediation analysis

Adjusting the regressions in our sensitivity analysis for BMI reveals that the associations between eating behaviours and metabolic markers at 16 years of life are potentially affected by concurrent BMI. In order to explore this further, we examine if any statistically significant associations discovered in the main analyses are explained by mediation through BMI measured intermediately, between the eating behaviours trajectories and the metabolites, at 12 years. For simplicity, we focus only on comparisons between significantly associated trajectory versus the corresponding reference trajectory (e.g., high persistent fussy eating versus low fussy eating). Direct, indirect and total associations are illustrated in Supplementary Fig. 4, and are approximated using the SEM package in Stata version 16.1, retaining the a priori specified *p* value threshold of 0.003.

## Supplementary Information

Supplementary Information 1.

Supplementary Information 2.

## Data Availability

This study is based on data from the ALSPAC study (http://www.bristol.ac.uk/alspac/). Interested researchers can apply for data access with the University of Bristol, UK. Analysis scripts can be requested from the authors.

## References

[1] Han JC, Lawlor DA, Kimm SYS (2010). Childhood obesity. Lancet.

[2] Reilly JJ, Kelly J (2011). Long-term impact of overweight and obesity in childhood and adolescence on morbidity and premature mortality in adulthood: systematic review. Int. J. Obes..

[3] Daly M, Sutin AR, Robinson E (2019). Perceived weight discrimination mediates the prospective association between obesity and physiological dysregulation: evidence from a population-based cohort. Psychol. Sci..

[4] Spahlholz J, Baer N, König H-H, Riedel-Heller SG, Luck-Sikorski C (2016). Obesity and discrimination—a systematic review and meta-analysis of observational studies. Obes. Rev..

[5] Bjerregaard LG (2018). Change in overweight from childhood to early adulthood and risk of type 2 diabetes. N. Engl. J. Med..

[6] Kelsey MM, Zaepfel A, Bjornstad P, Nadeau KJ (2014). Age-related consequences of childhood obesity. Gerontology.

[7] Halfon N, Larson K, Slusser W (2013). Associations between obesity and comorbid mental health, developmental, and physical health conditions in a nationally representative sample of US children aged 10 to 17. Acad. Pediatr..

[8] Ng M (2014). Global, regional, and national prevalence of overweight and obesity in children and adults during 1980–2013: a systematic analysis for the Global Burden of Disease Study 2013. Lancet.

[9] Goodarzi MO (2018). Genetics of obesity: what genetic association studies have taught us about the biology of obesity and its complications. Lancet Diabetes Endocrinol..

[10] Phillips CM (2017). Metabolically healthy obesity across the life course: epidemiology, determinants, and implications. Ann. N. Y. Acad. Sci..

[11] Swinburn BA (2011). The global obesity pandemic: shaped by global drivers and local environments. Lancet.

[12] Boswell N, Byrne R, Davies PSW (2018). Aetiology of eating behaviours: A possible mechanism to understand obesity development in early childhood. Neurosci. Biobehav. Rev..

[13] Konttinen H (2015). Appetitive traits as behavioural pathways in genetic susceptibility to obesity: A population-based cross-sectional study. Sci. Rep..

[14] Jacob R (2018). The role of eating behavior traits in mediating genetic susceptibility to obesity. Am. J. Clin. Nutr..

[15] Steinsbekk S, Wichstrøm L (2015). Predictors of change in BMI from the age of 4 to 8. J. Pediatr. Psychol..

[16] Parkinson KN, Drewett RF, Le Couteur AS, Adamson AJ, Gateshead Milennium Study Core Team (2010). Do maternal ratings of appetite in infants predict later Child Eating Behaviour Questionnaire scores and body mass index?. Appetite.

[17] Herle M (2020). Eating behavior trajectories in the first 10 years of life and their relationship with BMI. Int. J. Obes..

[18] de Barse LM (2015). Longitudinal association between preschool fussy eating and body composition at 6 years of age: The Generation R Study. Int. J. Behav. Nutr. Phys. Act..

[19] Cirulli ET (2019). Profound perturbation of the metabolome in obesity is associated with health risk. Cell Metab..

[20] Hellmuth C (2019). An individual participant data meta-analysis on metabolomics profiles for obesity and insulin resistance in European children. Sci. Rep..

[21] McCullough ML (2019). Metabolomic markers of healthy dietary patterns in US postmenopausal women. Am. J. Clin. Nutr..

[22] Pallister T (2016). Characterizing blood metabolomics profiles associated with self-reported food intakes in female twins. PLoS ONE.

[23] Scaglioni S (2018). Factors influencing children’s eating behaviours. Nutrients.

[24] Perng W (2014). Metabolomic profiles and childhood obesity. Obesity.

[25] McCormack SE (2013). Circulating branched-chain amino acid concentrations are associated with obesity and future insulin resistance in children and adolescents. Pediatr. Obes..

[26] Sarin HV (2019). Food neophobia associates with poorer dietary quality, metabolic risk factors, and increased disease outcome risk in population-based cohorts in a metabolomics study. Am. J. Clin. Nutr..

[27] Herle M (2019). A longitudinal study of eating behaviours in childhood and later eating disorder behaviours and diagnoses. Br. J. Psychiatry.

[28] Fraser A (2013). Cohort Profile: The Avon Longitudinal Study of Parents and Children: ALSPAC mothers cohort. Int. J. Epidemiol..

[29] Boyd A (2013). Cohort Profile: The ‘children of the 90s’–the index offspring of the Avon Longitudinal Study of Parents and Children. Int. J. Epidemiol..

[30] Varbo A, Freiberg JJ, Nordestgaard BG (2018). Remnant cholesterol and myocardial infarction in normal weight, overweight, and obese individuals from the Copenhagen General Population Study. Clin. Chem..

[31] Varbo A (2013). Remnant cholesterol as a causal risk factor for ischemic heart disease. J. Am. Coll. Cardiol..

[32] DeBerardinis RJ, Cheng T (2010). Q’s next: The diverse functions of glutamine in metabolism, cell biology and cancer. Oncogene.

[33] Lynch CJ, Adams SH (2014). Branched-chain amino acids in metabolic signalling and insulin resistance. Nat. Rev. Endocrinol..

[34] Saeed A (2018). Remnant-like particle cholesterol, low-density lipoprotein triglycerides, and incident cardiovascular disease. J. Am. Coll. Cardiol..

[35] Karpe F (1999). Postprandial lipoprotein metabolism and atherosclerosis. J. Intern. Med..

[36] Lawler, P. R. *et al.* Residual risk of atherosclerotic cardiovascular events in relation to reductions in very-low-density lipoproteins. *J. Am. Heart Assoc.* **6**(12), e007402 (2017). 10.1161/JAHA.117.007402 PMC5779048 29223956

[37] Sandesara PB, Virani SS, Fazio S, Shapiro MD (2019). The forgotten lipids: Triglycerides, remnant cholesterol, and atherosclerotic cardiovascular disease risk. Endocr. Rev..

[38] Varbo A, Nordestgaard BG (2019). Remnant cholesterol and risk of ischemic stroke in 112,512 individuals from the general population. Ann. Neurol..

[39] Goliasch G (2015). Premature myocardial infarction is strongly associated with increased levels of remnant cholesterol. J. Clin. Lipidol..

[40] Sniderman AD (2019). Apolipoprotein B particles and cardiovascular disease: A narrative review. JAMA Cardiol.

[41] Carmena R, Duriez P, Fruchart J-C (2004). Atherogenic lipoprotein particles in atherosclerosis. Circulation.

[42] Cromwell WC (2007). LDL particle number and risk of future cardiovascular disease in the Framingham offspring study—Implications for LDL management. J. Clin. Lipidol..

[43] Sniderman AD, Lamarche B, Contois JH, de Graaf J (2014). Discordance analysis and the Gordian Knot of LDL and non-HDL cholesterol versus apoB. Curr. Opin. Lipidol..

[44] Bell JD, Brown JCC, Nicholson JK, Sadler PJ (1987). Assignment of resonances for ‘acute-phase’glycoproteins in high resolution proton NMR spectra of human blood plasma. FEBS Lett..

[45] Dullaart RPF, Gruppen EG, Connelly MA, Otvos JD, Lefrandt JD (2015). GlycA, a biomarker of inflammatory glycoproteins, is more closely related to the leptin/adiponectin ratio than to glucose tolerance status. Clin. Biochem..

[46] Connelly MA, Otvos JD, Shalaurova I, Playford MP, Mehta NN (2017). GlycA, a novel biomarker of systemic inflammation and cardiovascular disease risk. J. Transl. Med..

[47] Akinkuolie AO, Pradhan AD, Buring JE, Ridker PM, Mora S (2015). Novel protein glycan side-chain biomarker and risk of incident type 2 diabetes mellitus. Arterioscler. Thromb. Vasc. Biol..

[48] Kettunen J (2018). Biomarker glycoprotein acetyls is associated with the risk of a wide spectrum of incident diseases and stratifies mortality risk in angiography patients. Circul. Genom. Precis. Med..

[49] Duprez DA, Otvos J, Sanchez OA, Mackey RH (2016). Comparison of the predictive value of GlycA and other biomarkers of inflammation for total death, incident cardiovascular events, noncardiovascular and noncancer inflammatory-related events, and total cancer events. Clin. Chem..

[50] Muhlestein JB (2018). GlycA and hsCRP are independent and additive predictors of future cardiovascular events among patients undergoing angiography: The intermountain heart collaborative study. Am. Heart J..

[51] Ivanova EA, Myasoedova VA, Melnichenko AA, Grechko AV, Orekhov AN (2017). Small dense low-density lipoprotein as biomarker for atherosclerotic diseases. Oxid. Med. Cell. Longev..

[52] Plourde M, Cunnane SC (2007). Extremely limited synthesis of long chain polyunsaturates in adults: Implications for their dietary essentiality and use as supplements. Appl. Physiol. Nutr. Metab..

[53] Shahidi F, Ambigaipalan P (2018). Omega-3 polyunsaturated fatty acids and their health benefits. Annu. Rev. Food Sci. Technol..

[54] Buczynski MW, Dumlao DS, Dennis EA (2009). Thematic review series: Proteomics. An integrated omics analysis of eicosanoid biology. J. Lipid Res..

[55] Dennis EA, Norris PC (2015). Eicosanoid storm in infection and inflammation. Nat. Rev. Immunol..

[56] Zárate R, El Jaber-Vazdekis N, Tejera N, Pérez JA, Rodríguez C (2017). Significance of long chain polyunsaturated fatty acids in human health. Clin. Transl. Med..

[57] Calder PC (2013). Omega-3 polyunsaturated fatty acids and inflammatory processes: nutrition or pharmacology?. Br. J. Clin. Pharmacol..

[58] Rizos EC, Ntzani EE, Bika E, Kostapanos MS, Elisaf MS (2012). Association between omega-3 fatty acid supplementation and risk of major cardiovascular disease events: a systematic review and meta-analysis. JAMA.

[59] O’Connell TD, Block RC, Huang SP, Shearer GC (2017). ω3-Polyunsaturated fatty acids for heart failure: Effects of dose on efficacy and novel signaling through free fatty acid receptor 4. J. Mol. Cell. Cardiol..

[60] Mozaffarian D, Wu JHY (2011). Omega-3 fatty acids and cardiovascular disease: Effects on risk factors, molecular pathways, and clinical events. J. Am. Coll. Cardiol..

[61] Hooper L (2006). Risks and benefits of omega 3 fats for mortality, cardiovascular disease, and cancer: Systematic review. BMJ.

[62] de Lorgeril M, Salen P, Defaye P, Rabaeus M (2013). Recent findings on the health effects of omega-3 fatty acids and statins, and their interactions: Do statins inhibit omega-3?. BMC Med..

[63] Bowen KJ, Harris WS, Kris-Etherton PM (2016). Omega-3 fatty acids and cardiovascular disease: Are there benefits?. Curr. Treat. Options Cardiovasc. Med..

[64] Simopoulos AP (2016). An increase in the Omega-6/Omega-3 fatty acid ratio increases the risk for obesity. Nutrients.

[65] Schuchardt JP (2011). Incorporation of EPA and DHA into plasma phospholipids in response to different omega-3 fatty acid formulations—a comparative bioavailability study of fish oil vs. krill oil. Lipids Health Dis..

[66] Costello, L. C. & Franklin, R. B. Plasma citrate homeostasis: how it is regulated; and its physiological and clinical implications. An important, but neglected, relationship in medicine. *HSOA J. Hum. Endocrinol.* **1**(1), 005 (2016). PMC5345696 28286881

[67] Akram M (2014). Citric acid cycle and role of its intermediates in metabolism. Cell Biochem. Biophys..

[68] Iacobazzi V, Infantino V (2014). Citrate–new functions for an old metabolite. Biol. Chem..

[69] Fischer B (2015). Inverse relationship between body mass index and mitochondrial oxidative phosphorylation capacity in human subcutaneous adipocytes. Am. J. Physiol. Endocrinol. Metab..

[70] Christe M (2013). Obesity affects mitochondrial citrate synthase in human omental adipose tissue. ISRN Obes.

[71] Zamora-Mendoza R (2018). Dysregulation of mitochondrial function and biogenesis modulators in adipose tissue of obese children. Int. J. Obes..

[72] Durante W (2019). The emerging role of l-glutamine in cardiovascular health and disease. Nutrients.

[73] Cheng S (2012). Metabolite profiling identifies pathways associated with metabolic risk in humans. Circulation.

[74] Ma W (2018). Dietary glutamine, glutamate and mortality: Two large prospective studies in US men and women. Int. J. Epidemiol..

[75] Guasch-Ferré M (2016). Metabolomics in prediabetes and diabetes: A systematic review and meta-analysis. Diabetes Care.

[76] Würtz P (2012). Metabolic signatures of insulin resistance in 7,098 young adults. Diabetes.

[77] Greenfield JR (2009). Oral glutamine increases circulating glucagon-like peptide 1, glucagon, and insulin concentrations in lean, obese, and type 2 diabetic subjects. Am. J. Clin. Nutr..

[78] Mansour A (2015). Effect of glutamine supplementation on cardiovascular risk factors in patients with type 2 diabetes. Nutrition.

[79] Brosnan JT, Brosnan ME (2006). Branched-chain amino acids: Enzyme and substrate regulation. J. Nutr..

[80] Burrage LC, Nagamani SCS, Campeau PM, Lee BH (2014). Branched-chain amino acid metabolism: From rare Mendelian diseases to more common disorders. Hum. Mol. Genet..

[81] Tobias DK, Mora S, Verma S, Lawler PR (2018). Altered branched chain amino acid metabolism: Toward a unifying cardiometabolic hypothesis. Curr. Opin. Cardiol..

[82] Wang TJ (2011). Metabolite profiles and the risk of developing diabetes. Nat. Med..

[83] Newgard CB (2009). A branched-chain amino acid-related metabolic signature that differentiates obese and lean humans and contributes to insulin resistance. Cell Metab..

[84] Zhao X (2016). The relationship between branched-chain amino acid related metabolomic signature and insulin resistance: A systematic review. J. Diabetes Res..

[85] Boulet MM (2015). Alterations of plasma metabolite profiles related to adipose tissue distribution and cardiometabolic risk. Am. J. Physiol. Endocrinol. Metab..

[86] Mangge H (2016). Branched-chain amino acids are associated with cardiometabolic risk profiles found already in lean, overweight and obese young. J. Nutr. Biochem..

[87] Cummings JH (1981). Short chain fatty acids in the human colon. Gut.

[88] Akanji AO, Humphreys S, Thursfield V, Hockaday TD (1989). The relationship of plasma acetate with glucose and other blood intermediary metabolites in non-diabetic and diabetic subjects. Clin. Chim. Acta.

[89] Scheppach W, Pomare EW, Elia M, Cummings JH (1991). The contribution of the large intestine to blood acetate in man. Clin. Sci..

[90] Fernandes J, Vogt J, Wolever TMS (2014). Kinetic model of acetate metabolism in healthy and hyperinsulinaemic humans. Eur. J. Clin. Nutr..

[91] Chambers ES, Morrison DJ, Frost G (2015). Control of appetite and energy intake by SCFA: What are the potential underlying mechanisms?. Proc. Nutr. Soc..

[92] Frost G (2014). The short-chain fatty acid acetate reduces appetite via a central homeostatic mechanism. Nat. Commun..

[93] Lim J, Henry CJ, Haldar S (2016). Vinegar as a functional ingredient to improve postprandial glycemic control-human intervention findings and molecular mechanisms. Mol. Nutr. Food Res..

[94] Fernandes J, Vogt J, Wolever TMS (2012). Intravenous acetate elicits a greater free fatty acid rebound in normal than hyperinsulinaemic humans. Eur. J. Clin. Nutr..

[95] Layden BT, Yalamanchi SK, Wolever TM, Dunaif A, Lowe WL (2012). Negative association of acetate with visceral adipose tissue and insulin levels. Diabetes Metab. Syndr. Obes..

[96] Crouse JR, Gerson CD, DeCarli LM, Lieber CS (1968). Role of acetate in the reduction of plasma free fatty acids produced by ethanol in man. J. Lipid Res..

[97] Wolever TM, Spadafora P, Eshuis H (1991). Interaction between colonic acetate and propionate in humans. Am. J. Clin. Nutr..

[98] Playdon MC (2016). Comparing metabolite profiles of habitual diet in serum and urine. Am. J. Clin. Nutr..

[99] Lau C-HE (2018). Determinants of the urinary and serum metabolome in children from six European populations. BMC Med..

[100] Kochhar S (2006). Probing gender-specific metabolism differences in humans by nuclear magnetic resonance-based metabonomics. Anal. Biochem..

[101] Ellul S (2020). Sex differences in infant blood metabolite profile in association with weight and adiposity measures. Pediatr. Res..

[102] Dunn WB, Broadhurst DI, Atherton HJ, Goodacre R, Griffin JL (2011). Systems level studies of mammalian metabolomes: The roles of mass spectrometry and nuclear magnetic resonance spectroscopy. Chem. Soc. Rev..

[103] Haid M (2018). Long-term stability of human plasma metabolites during storage at -80 °C. J. Proteome Res..

[104] Tikkanen, E. *et al.* Metabolic biomarkers for peripheral artery disease compared with coronary artery disease: Lipoprotein and metabolite profiling of 31,657 individuals from five prospective cohorts. *medRxiv* 2020.07.24.20158675 (2020). 10.1161/JAHA.121.021995 PMC9075369 34845932

[105] Kettunen J (2016). Genome-wide study for circulating metabolites identifies 62 loci and reveals novel systemic effects of LPA. Nat. Commun..

[106] Holmes MV (2018). Lipids, lipoproteins, and metabolites and risk of myocardial infarction and stroke. J. Am. Coll. Cardiol..

[107] Battram T (2018). Coronary artery disease, genetic risk and the metabolome in young individuals. Wellcome Open Res..

[108] Syrad, H., Johnson, L., Wardle, J., & Llewellyn, C. H. (2016). Appetitive traits and food intake patterns in early life. *The American Journal of Clinical Nutrition*, **103**(1), 231–235. 10.3945/ajcn.115.117382 PMC4691671 26675767

[109] Daniels LA (2014). Child eating behavior outcomes of an early feeding intervention to reduce risk indicators for child obesity: The NOURISH RCT. Obesity.

[110] Golding J, Pembrey M, Jones R, ALSPAC Study Team (2001). ALSPAC—the Avon Longitudinal Study of Parents and Children. I. Study methodology. Paediatr. Perinat. Epidemiol..

[111] Birmingham K (2018). Pioneering Ethics in a Longitudinal Study.

[112] Nagin DS, Odgers CL (2010). Group-based trajectory modeling (nearly) two decades later. J. Quant. Criminol..

[113] Herle M (2020). Identifying typical trajectories in longitudinal data: Modelling strategies and interpretations. Eur. J. Epidemiol..

[114] Soininen P (2009). High-throughput serum NMR metabonomics for cost-effective holistic studies on systemic metabolism. Analyst.

[115] Moyano D, Vilaseca MA, Artuch R, Lambruschini N (1998). Plasma amino acids in anorexia nervosa. Eur. J. Clin. Nutr..

[116] Palova S, Charvat J, Masopust J, Klapkova E, Kvapil M (2007). Changes in the plasma amino acid profile in anorexia nervosa. J. Int. Med. Res..

[117] Würtz P (2017). Quantitative serum nuclear magnetic resonance metabolomics in large-scale epidemiology: A primer on Omic Technologies. Am. J. Epidemiol..

[118] Wang Q (2018). Metabolic characterization of menopause: Cross-sectional and longitudinal evidence. BMC Med..

[119] Wang Q (2016). Metabolic profiling of pregnancy: Cross-sectional and longitudinal evidence. BMC Med..

[120] Santos Ferreira DL (2019). The effect of pre-analytical conditions on blood metabolomics in epidemiological studies. Metabolites.

[121] Santos Ferreira DL (2017). Association of pre-pregnancy body mass index with offspring metabolic profile: Analyses of 3 European prospective birth cohorts. PLoS Med..

[122] Taylor K (2020). Metabolomics datasets in the Born in Bradford cohort. Wellcome Open Res..

[123] Sliz E (2018). Metabolomic consequences of genetic inhibition of PCSK9 compared with statin treatment. Circulation.

[124] McBride N (2020). Do nuclear magnetic resonance (NMR)-based metabolomics improve the prediction of pregnancy-related disorders? Findings from a UK birth cohort with independent validation. BMC Med..

[125] Würtz P (2015). Metabolite profiling and cardiovascular event risk: A prospective study of 3 population-based cohorts. Circulation.

