# Supplementary material for: Childhood overeating is associated with adverse cardiometabolic and inflammatory profiles in adolescence
Source: Sci Rep. 2021 Jun 14;11:12478. doi: 10.1038/s41598-021-90644-2 (PMC8203659; doi:10.1038/s41598-021-90644-2)
Supplement: Supplementary file 2 — Supplementary Information 2. [file 41598_2021_90644_MOESM2_ESM.pdf]

Supplementary Methods and Figures

Childhood overeating is associated with adverse cardiometabolic and inflammatory profiles in adolescence

Authors: Christopher Hübel[1,2,3,4]*, Moritz Herle[5]*, Diana L. Santos Ferreira[6,7], Mohamed Abdulkadir[8,9], Rachel Bryant-Waugh[10], Ruth J. F. Loos[11], Cynthia M. Bulik[4,12,13], Deborah A. Lawlor[6,7], Nadia Micali[8,9,14,15]

[1]Social, Genetic & Developmental Psychiatry Centre, Institute of Psychiatry, Psychology & Neuroscience, King's College London, UK
[2]UK National Institute for Health Research (NIHR) Biomedical Research Centre for Mental Health, South London and Maudsley Hospital, London, UK
[3]National Centre for Register-based Research, Department of Economics and Business Economics, Aarhus University, Aarhus, Denmark
[4]Department of Medical Epidemiology and Biostatistics, Karolinska Institutet, Stockholm, Sweden
[5]Department of Biostatistics & Health Informatics, Institute of Psychiatry, Psychology & Neuroscience, King's College London, UK
[6]Medical Research Council Integrative Epidemiology Unit at the University of Bristol, Bristol, UK
[7]Population Health Sciences, Bristol Medical School, University of Bristol, Bristol, UK
[8]Department of Pediatrics Gynaecology and Obstetrics, Faculty of Medicine, University of Geneva, Geneva, Switzerland
[9]Department of Psychiatry, Faculty of Medicine, University of Geneva, Geneva, Switzerland
[10]Maudsley Centre for Child and Adolescent Eating Disorders, Michael Rutter Centre for Children and Young People, Maudsley Hospital, London, UK
[11]Icahn School of Medicine at Mount Sinai, New York, New York, USA
[12]Department of Psychiatry, University of North Carolina at Chapel Hill, Chapel Hill, NC, USA
[13]Department of Nutrition, University of North Carolina at Chapel Hill, Chapel Hill, NC, USA
[14]Bristol National Institute of Health Research Biomedical Research Centre, Bristol, UK
[15]Great Ormond Street Institute of Child Health, University College London, London, UK

Content

Supplementary Figure 1a-c. Lipoprotein subclasses of the overeating analysis

Supplementary Figure 2a-c. Lipoprotein subclasses of the undereating analysis

Supplementary Figure 3a-c. Lipoprotein subclasses of the fussy eating analysis

Supplementary Figure 4. Conceptual diagram for mediation analyses

Supplementary Methods

Lipoprotein subclasses and fatty acids details

LIPO window

Supplementary Table S5. NMR metabolic measures

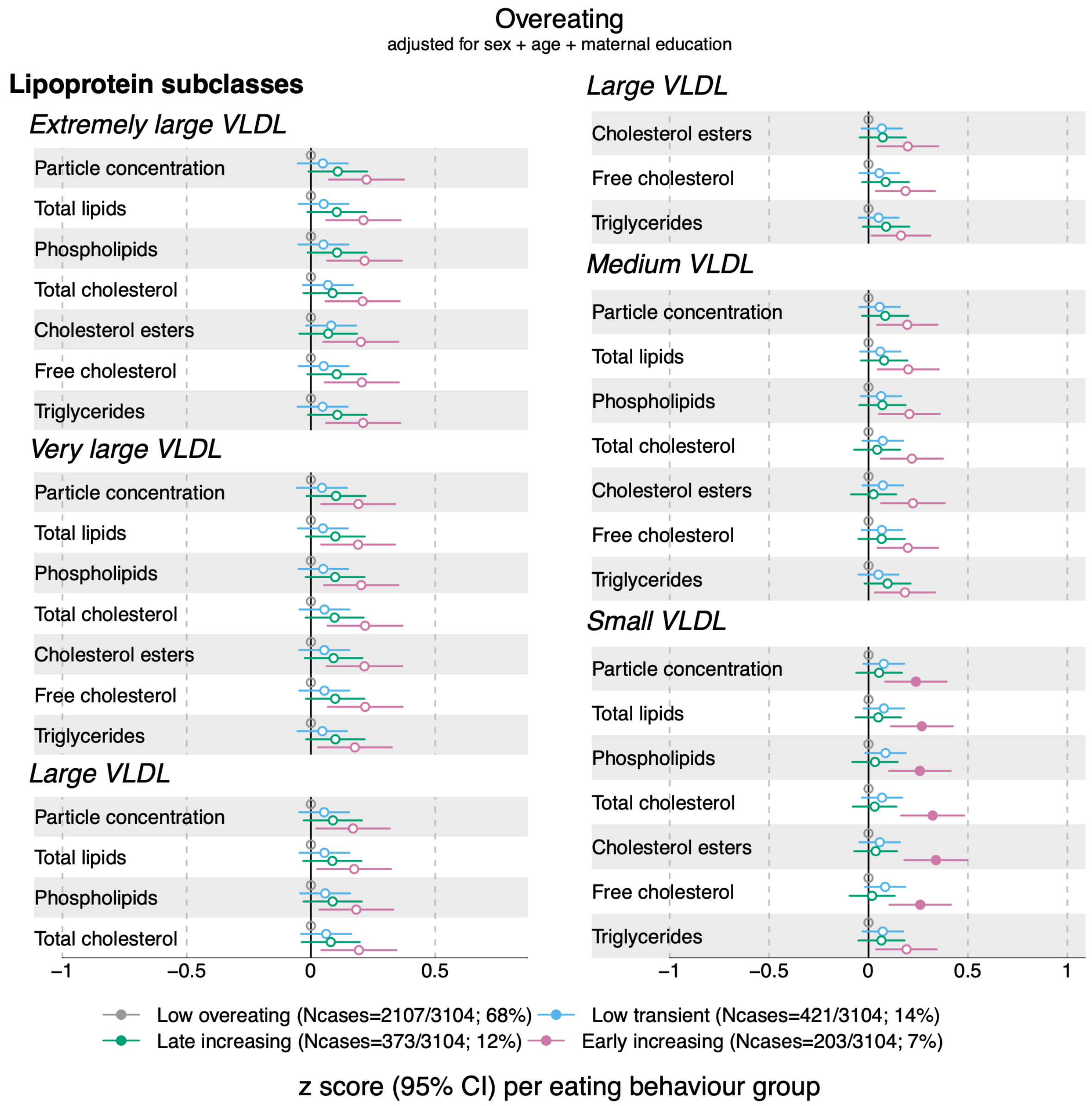

Supplementary Figure 1a. *Estimates refer to change in standardised metabolic trait concentration at 16 years in each overeating trajectory, in reference to the "low overeating" trajectory (gray dot). Error bars = 95% confidence intervals (CI). Analyses adjusted for sex, age at metabolite measure and maternal education. Note: Association where CIs do not cross 0, p-value <0.05; Filled dots: Association meets the p-value threshold of <0.003*

VLDL = very low-density lipoprotein

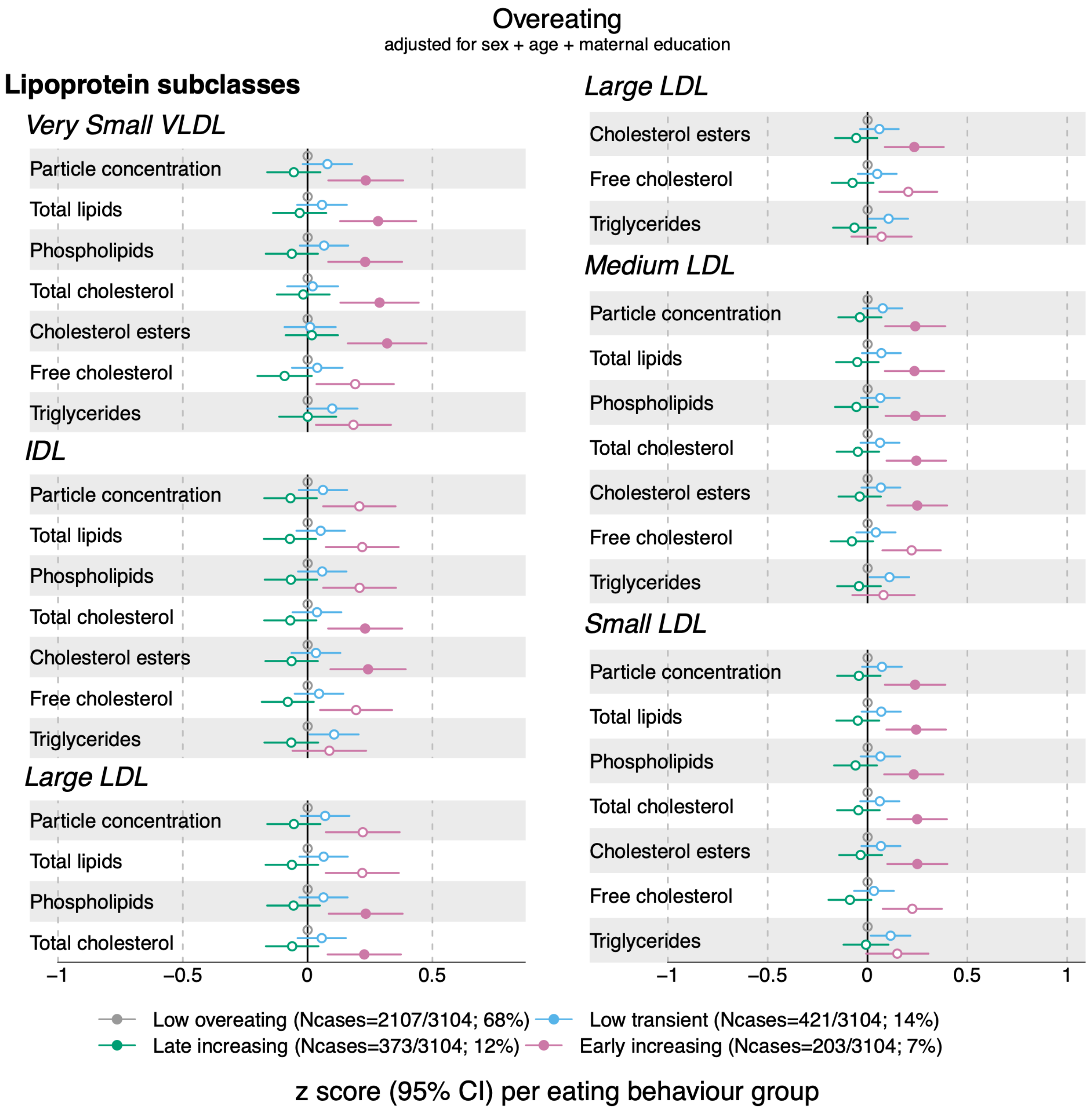

Supplementary Figure 1b. *Estimates refer to change in standardised metabolic trait concentration at 16 years in each overeating trajectory, in reference to the "low overeating" trajectory (gray dot). Error bars = 95% confidence intervals (CI). Analyses adjusted for sex, age at metabolite measure and maternal education. Note: Association where CIs do not cross 0, p-value <0.05; Filled dots: Association meets the p-value threshold of <0.003*

VLDL = very low-density lipoprotein, IDL = intermediate-density lipoprotein, LDL = low-density lipoprotein

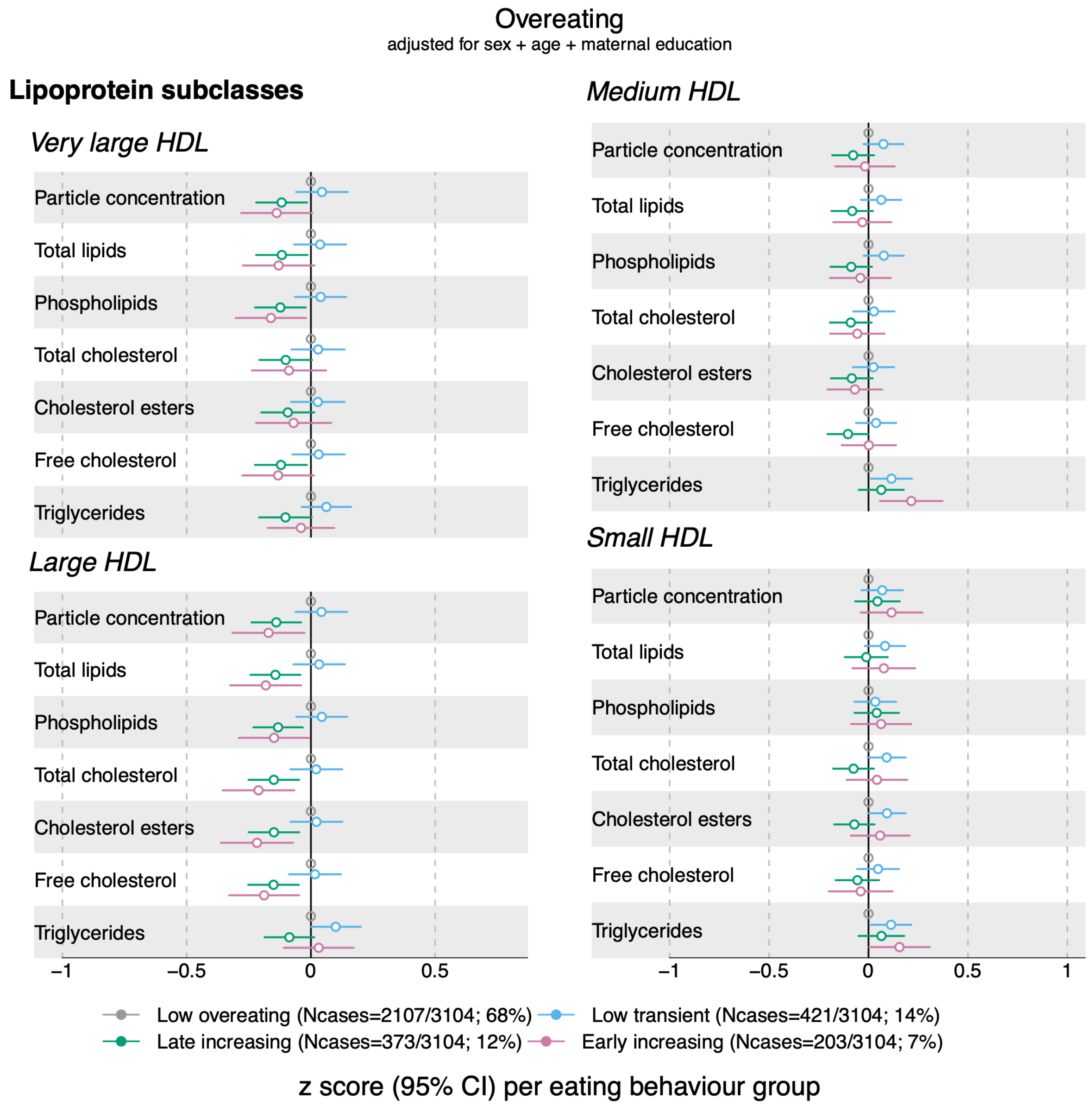

Supplementary Figure 1c. *Estimates refer to change in standardised metabolic trait concentration at 16 years in each overeating trajectory, in reference to the "low overeating" trajectory (gray dot). Error bars = 95% confidence intervals (CI). Analyses adjusted for sex, age at metabolite measure and maternal education. Note: Association where CIs do not cross 0, p-value <0.05; Filled dots: Association meets the p-value threshold of <0.003.*

HDL = high-density lipoprotein

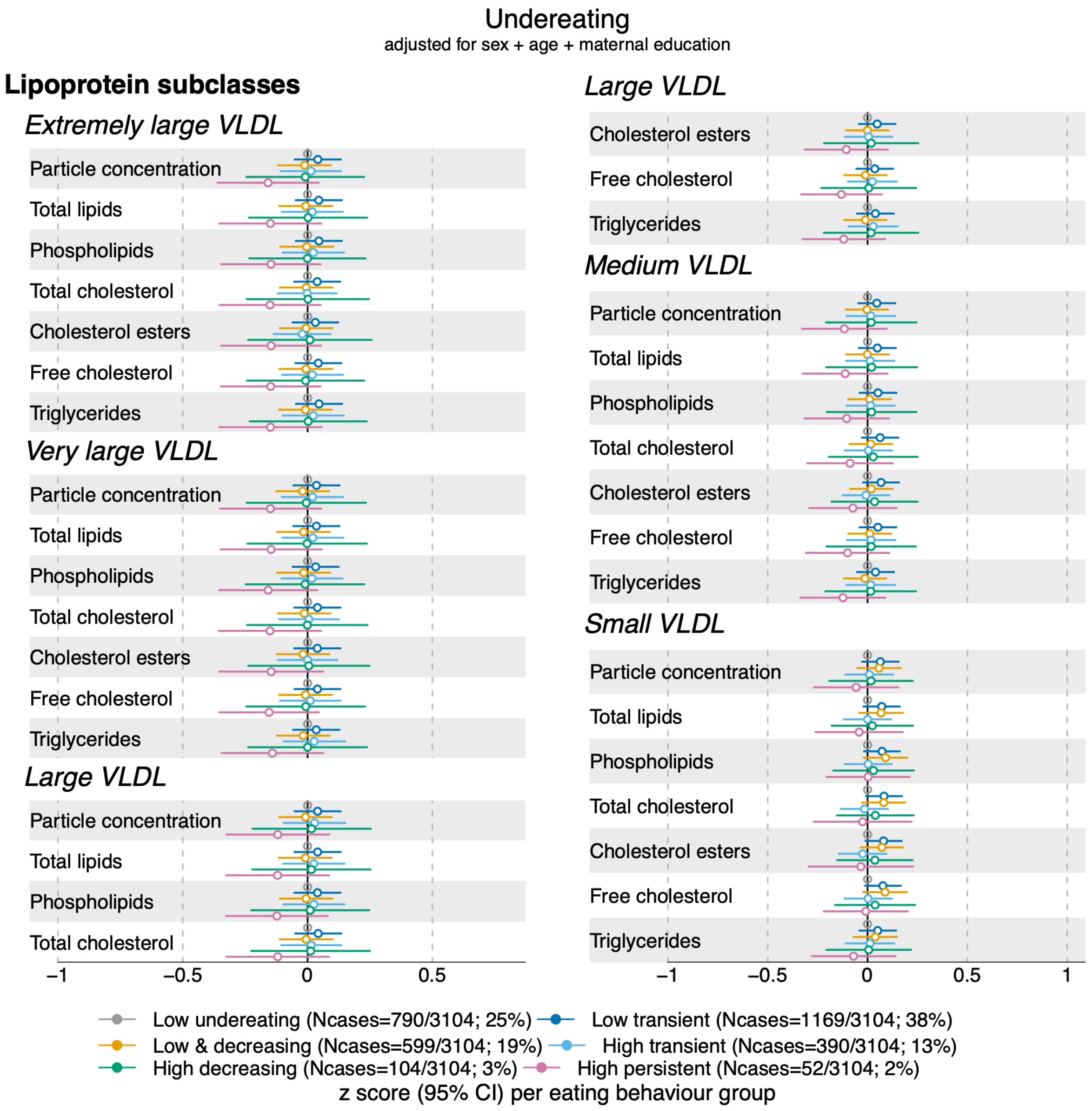

Supplementary Figure 2a. *Estimates refer to change in standardised metabolic trait concentration at 16 years in each undereating trajectory, in reference to the "low undereating" trajectory (gray dot). Error bars = 95% confidence intervals (CI). Analyses adjusted for sex, age at metabolite measure and maternal education. Note: Association where CIs do not cross 0, p-value <0.05; Filled dots: Association meets the p-value threshold of <0.003*

VLDL = very low-density lipoprotein

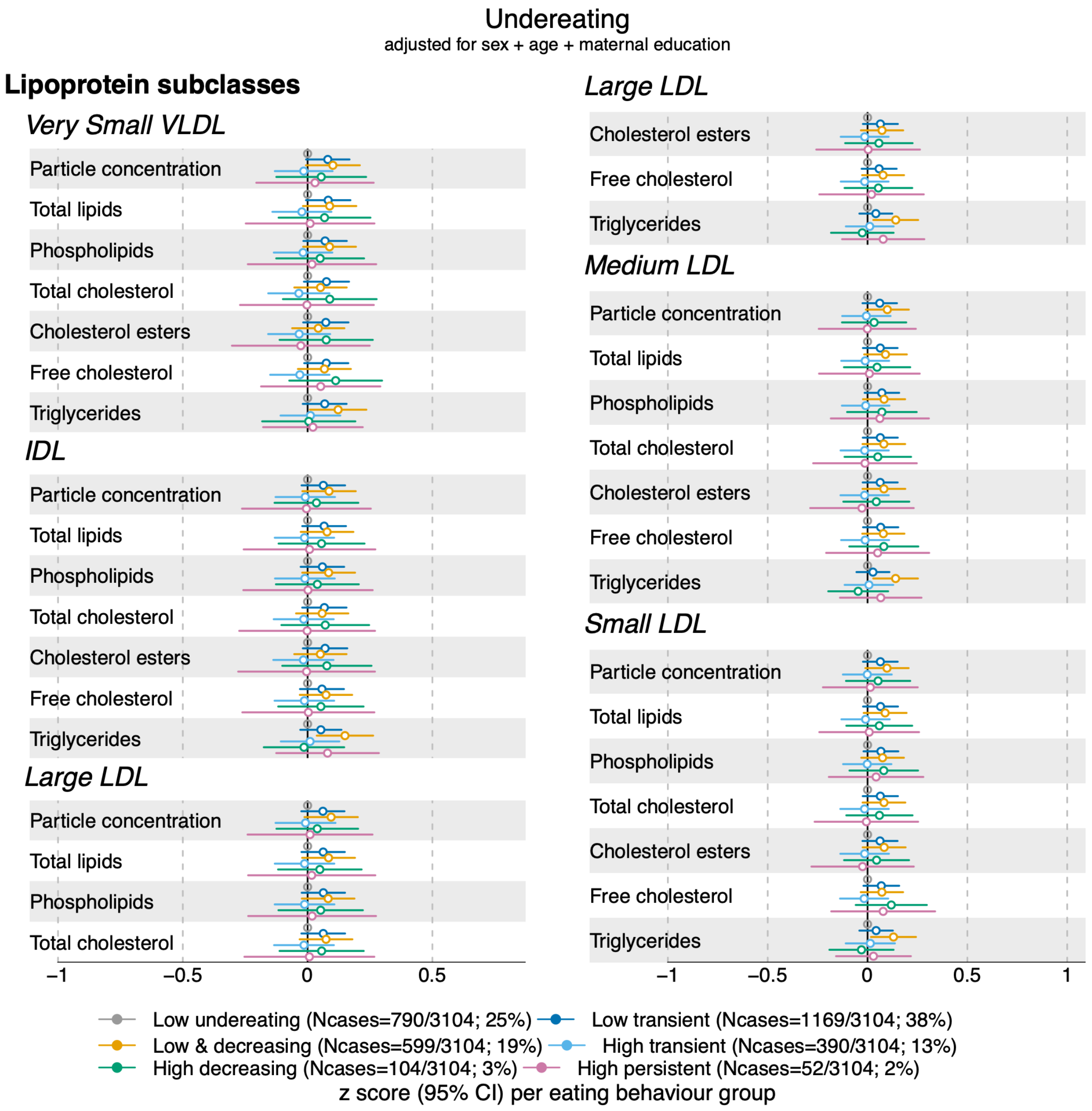

Supplementary Figure 2b. *Estimates refer to change in standardised metabolic trait concentration at 16 years in each undereating trajectory, in reference to the "low undereating" trajectory (gray dot). Error bars = 95% confidence intervals (CI). Analyses adjusted for sex, age at metabolite measure and maternal education. Note: Association where CIs do not cross 0, p-value <0.05; Filled dots: Association meets the p-value threshold of <0.003*

VLDL = very low-density lipoprotein, IDL = intermediate-density lipoprotein, LDL = low-density lipoprotein

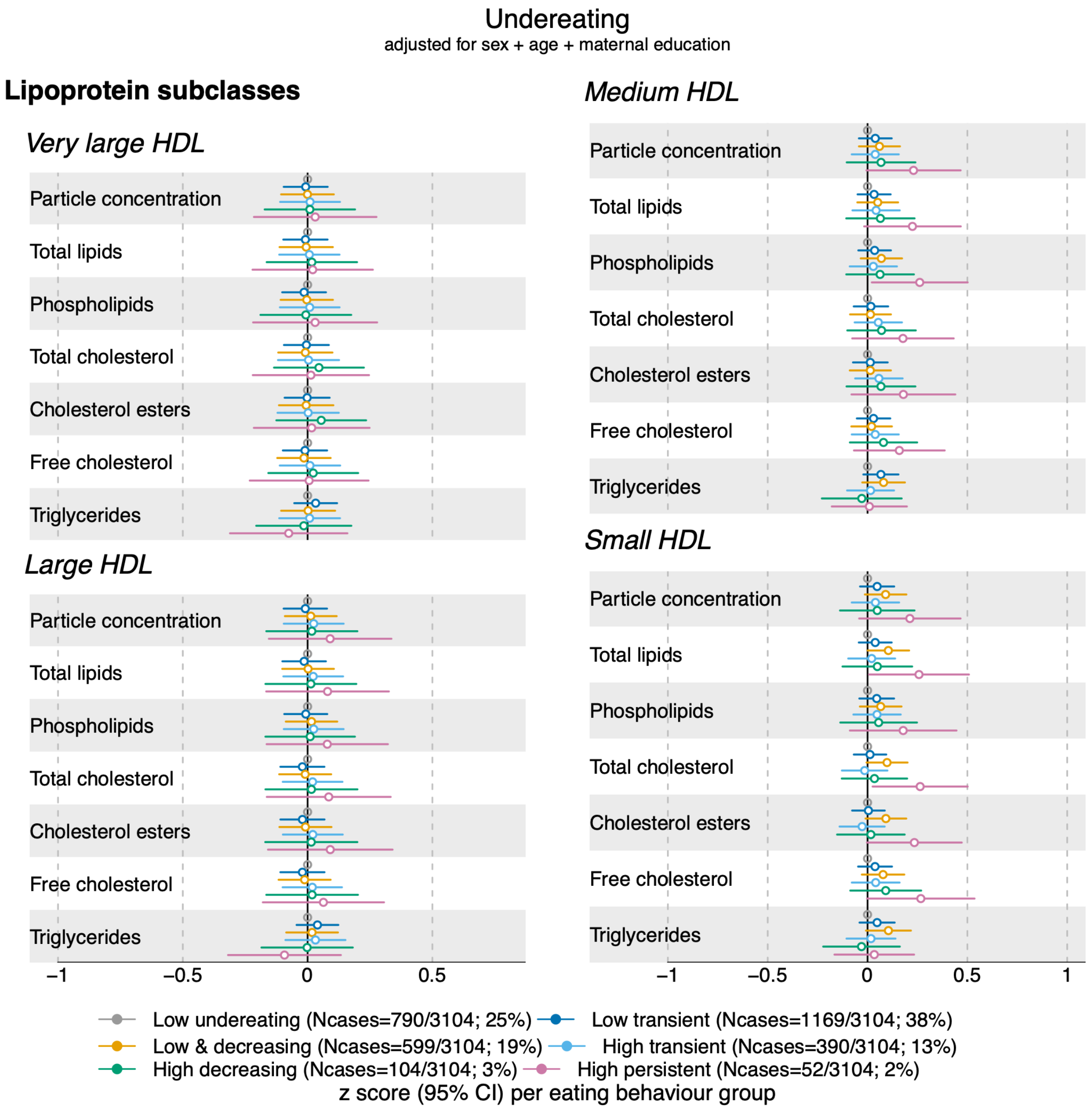

Supplementary Figure 2c. *Estimates refer to change in standardised metabolic trait concentration at 16 years in each undereating trajectory, in reference to the "low undereating" trajectory (gray dot). Error bars = 95% confidence intervals (CI). Analyses adjusted for sex, age at metabolite measure and maternal education. Note: Association where CIs do not cross 0, p-value <0.05; Filled dots: Association meets the p-value threshold of <0.003*

HDL = high-density lipoprotein

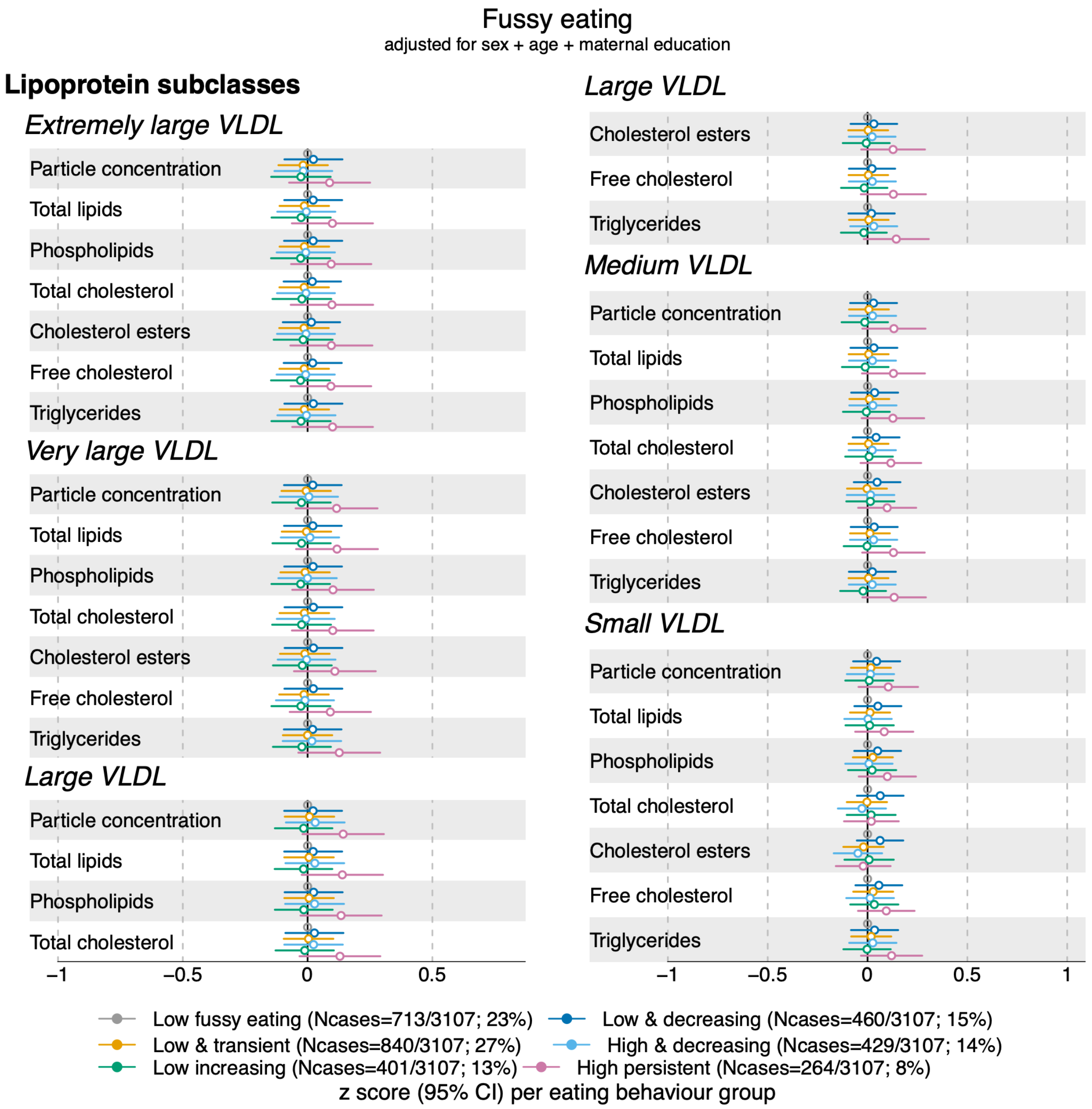

Supplementary Figure 3a. *Estimates refer to change in standardised metabolic trait concentration at 16 years in each fussy eating trajectory, in reference to the "low fussy eating" trajectory (gray dot). Error bars = 95% confidence intervals (CI). Analyses adjusted for sex, age at metabolite measure and maternal education. Note: Association where CIs do not cross 0, p-value <0.05; Filled dots: Association meets the p-value threshold of <0.003*

VLDL = very low-density lipoprotein

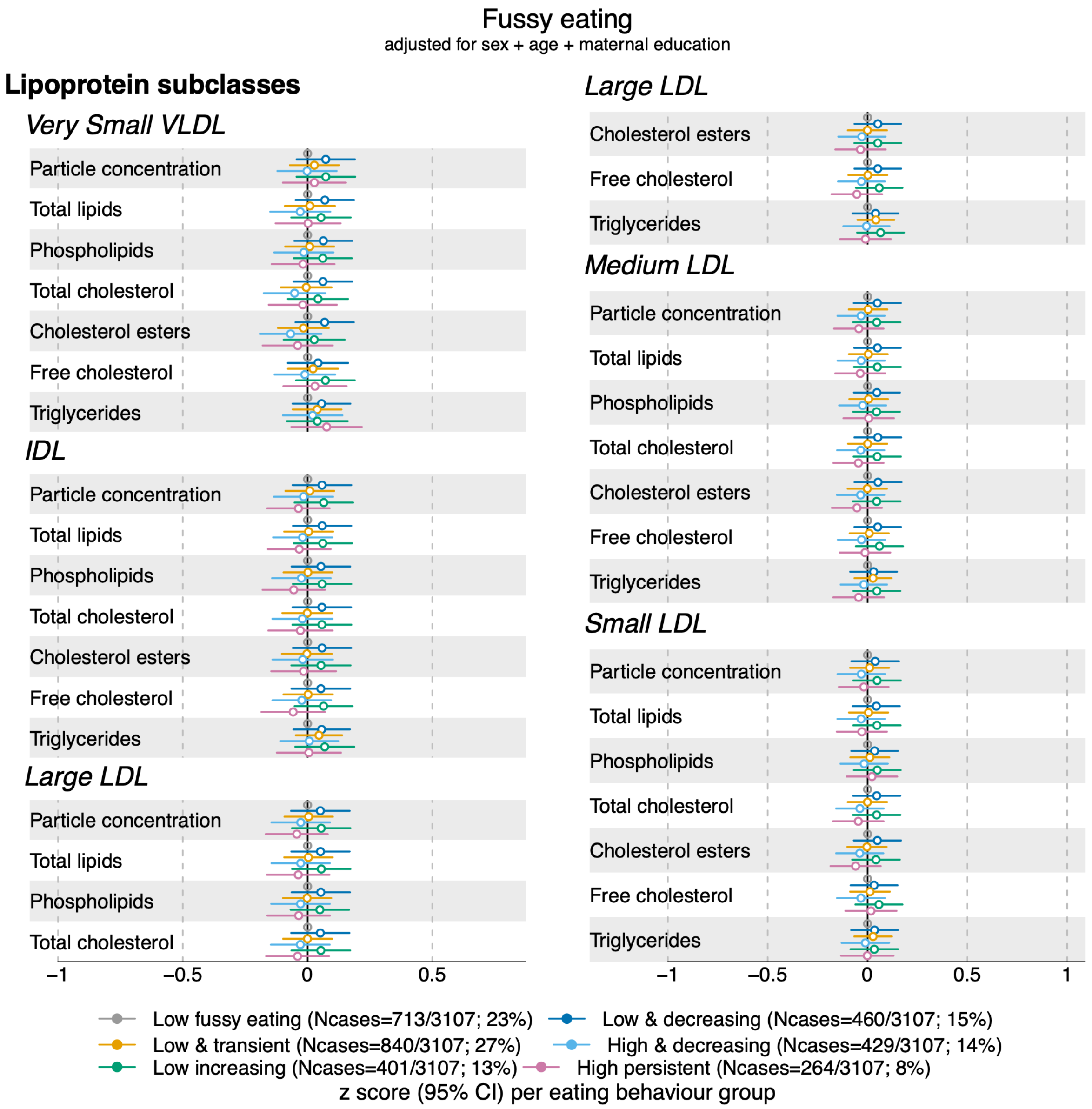

Supplementary Figure 3b. *Estimates refer to change in standardised metabolic trait concentration at 16 years in each fussy eating trajectory, in reference to the "low fussy eating" trajectory (gray dot). Error bars = 95% confidence intervals (CI). Analyses adjusted for sex, age at metabolite measure and maternal education. Note: Association where CIs do not cross 0, p-value <0.05; Filled dots: Association meets the p-value threshold of <0.003*

VLDL = very low-density lipoprotein, IDL = intermediate-density lipoprotein, LDL = low-density lipoprotein

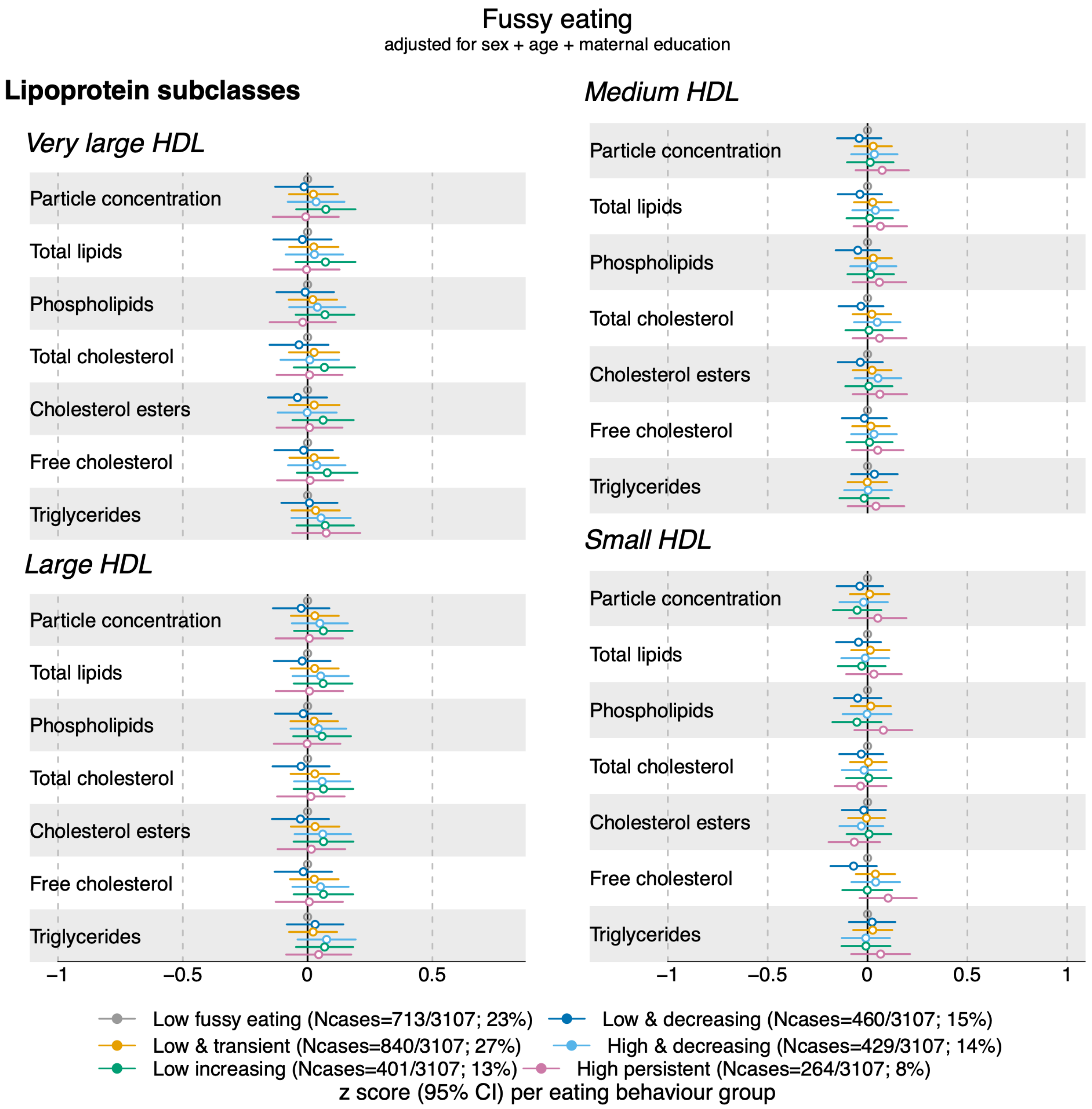

Supplementary Figure 3c. *Estimates refer to change in standardised metabolic trait concentration at 16 years in each fussy eating trajectory, in reference to the "low fussy eating" trajectory (gray dot). Error bars = 95% confidence intervals (CI). Analyses adjusted for sex, age at metabolite measure and maternal education. Note: Association where CIs do not cross 0, p-value <0.05; Filled dots: Association meets the p-value threshold of <0.003*

HDL = high-density lipoprotein

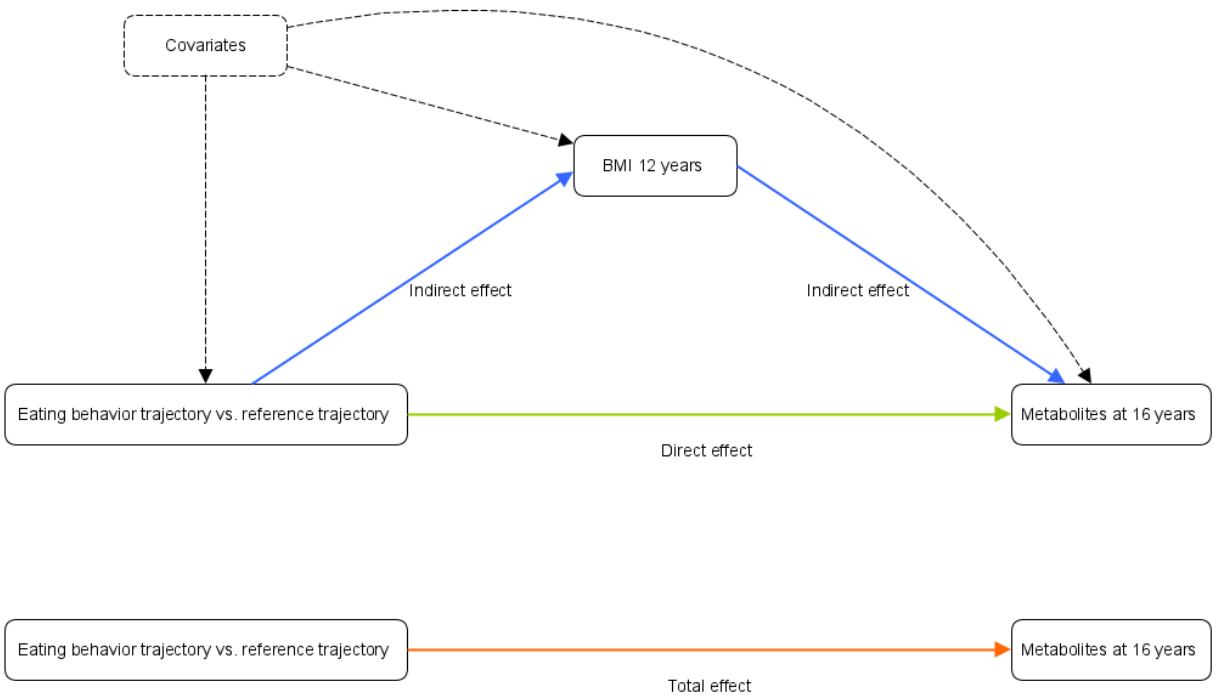

Supplementary Figure 4. *Conceptual diagram for mediation analyses illustrating direct, indirect, and total association.*

Supplementary Methods

Lipoprotein subclasses and fatty acids details

Each lipoprotein measurement is characterized by three elements: size (e.g., extremely large, very large, large, medium, small, very small), density (e.g., very low density lipoprotein [VLDL], intermediate density lipoprotein [IDL], low density lipoprotein [LDL], high density lipoprotein [HDL]) and trait (e.g., particle concentration, total lipids, triglycerides, phospholipids, total cholesterol, cholesterol esters, free cholesterol). The definition of the 14 lipoprotein subclass are as follows: six subclasses of VLDL: extremely large VLDL (particle diameters from 75 nm upwards and possible chylomicrons contributions), alongside 5 other VLDL subclasses (average particle diameters of 64.0 nm, 53.6 nm, 44.5 nm, 36.8 nm, and 31.3 nm); IDL (28.6 nm), 3 LDL subclasses (25.5 nm, 23.0 nm, and 18.7 nm), and 4 HDL subclasses (14.3 nm, 12.1 nm, 10.9 nm, and 8.7 nm). The lipoprotein traits obtained are the concentration of the lipoprotein size-density-trait combination in the total serum sample. For example, 0.5 mmol/l of very large VLDL cholesterol means 0.5 mmol of cholesterol embedded in very large VLDL particles per liter of serum or EDTA plasma. Remnant cholesterol was defined as VLDL cholesterol + IDL cholesterol, which is equivalent to total cholesterol (HDL cholesterol + LDL cholesterol)[1]. For fatty acids (FA), only the cis configuration was quantified since the trans fatty acids are below the platform's detection limit[1].

LIPO window

The LIPO window represents a standard spectrum of human serum displaying broad overlapping resonances arising from lipid molecules in various lipoprotein particles. The LIPO data are recorded using 8 transients acquired using a NOESY-presat pulse sequence with mixing time of 10ms and water peak suppression. The LMWM window includes signals from various low-molecular-weight molecules. The LMWM spectrum is recorded using a relaxation-filtered pulse sequence that suppresses most of the broad macromolecule and lipid signals to enhance detection of small solutes. Specifically, a Carr-Purcell-Meiboom-Gill (CPMG) pulse sequence with a 78ms T2-filter and fixed echo delay of 403μs is applied using 24 transients. The LIPID window of the serum extracts is acquired with a standard 1D spectrum using 32 transients.

Table S5: NMR metabolic measures

Molecular class	***Lipid, lipoprotein or metabolite name***	***Units***
Extremely large VLDL	*Concentration of chylomicrons and extremely large VLDL particles*	*mol/l*
	Total lipids in chylomicrons and extremely large VLDL	*mmol/l*
	Phospholipids in chylomicrons and extremely large VLDL	*mmol/l*
	Total cholesterol in chylomicrons and extremely large VLDL	*mmol/l*
	Free cholesterol in chylomicrons and extremely large VLDL	*mmol/l*
	Triglycerides in chylomicrons and extremely large VLDL	*mmol/l*
Very large VLDL	*Concentration of very large VLDL particles*	*mol/l*
	Total lipids in very large VLDL	*mmol/l*
	Phospholipids in very large VLDL	*mmol/l*
	Total cholesterol in very large VLDL	*mmol/l*
	Cholesterol esters in very large VLDL	*mmol/l*
	Free cholesterol in very large VLDL	*mmol/l*
	Triglycerides in very large VLDL	*mmol/l*
Large VLDL	*Concentration of large VLDL particles*	*mol/l*

	Total lipids in large VLDL	*mmol/l*
	Phospholipids in large VLDL	*mmol/l*
	Total cholesterol in large VLDL	*mmol/l l*
	Cholesterol esters in large VLDL	*mmol/l*
	Free cholesterol in large VLDL	*mmol/l*
	Triglycerides in large VLDL	*mmol/l*
Medium VLDL	*Concentration of large VLDL particles*	*mol/l*
	Total lipids in small VLDL	*mmol/l*
	Phospholipids in small VLDL	*mmol/l*
	Total cholesterol in small VLDL	*mmol/l*
	Cholesterol esters in small VLDL	*mmol/l*
	Free cholesterol in small VLDL	*mmol/l*
	Triglycerides in small VLDL	*mmol/l*
Small VLDL	*Concentration of very small VLDL particles*	*mol/l*
	Total lipids in very small VLDL	*mmol/l*
	Phospholipids in very small VLDL	*mmol/l*
	Total cholesterol in very small VLDL	*mmol/l*
	Cholesterol esters in very small VLDL	*mmol/l*
	Free cholesterol in very small VLDL	*mmol/l*

	Triglycerides in very small VLDL	*mmol/l*
IDl	*Concentration of IDL particles*	*mol/l*
	Total lipids in IDL	*mmol/l*
	Phospholipids in IDL	*mmol/l*
	Total cholesterol in IDL	*mmol/l*
	Cholesterol esters in IDL	*mmol/l*
	Free cholesterol in IDL	*mmol/l*
	Triglycerides in IDL	*mmol/l*
Large LDL	*Concentration of large LDL particles*	*mol/l*
	Total lipids in large LDL	*mmol/l*
	Phospholipids in large LDL	*mmol/l*
	Total cholesterol in large LDL	*mmol/l*
	Cholesterol esters in large LDL	*mmol/l*
	Free cholesterol in large LDL	*mmol/l*
	Triglycerides in large LDL	*mmol/l*
Medium LDL	*Concentration of medium LDL particles*	*mol/l*
	Total lipids in medium LDL	*mmol/l*
	Phospholipids in medium LDL	*mmol/l*
	Total cholesterol in medium LDL	*mmol/l*

	Cholesterol esters in medium LDL	*mmol/l*
	Free cholesterol in medium LDL	*mmol/l*
	Triglycerides in medium LDL	*mmol/l*
Small LDL	*Concentration of small LDL particles*	*mol/l*
	Total lipids in small LDL	*mmol/l*
	Phospholipids in small LDL	*mmol/l*
	Total cholesterol in small LDL	*mmol/l*
	Cholesterol esters in small LDL	*mmol/l*
	Free cholesterol in small LDL	*mmol/l*
	Triglycerides in small LDL	*mmol/l*
Very large HDL	*Concentration of very large HDL particles*	*mol/l*
	Total lipids in very large HDL	*mmol/l*
	Phospholipids in very large HDL	*mmol/l*
	Total cholesterol in very large HDL	*mmol/l*
	Cholesterol esters in very large HDL	*mmol/l*
	Free cholesterol in very large HDL	*mmol/l*
	Triglycerides in very large HDL	*mmol/l*
Large HDL	*Concentration of large HDL particles*	*mol/l*
	Total lipids in large HDL	*mmol/l*

	Phospholipids in large HDL	*mmol/l*
	Total cholesterol in large HDL	*mmol/l*
	Cholesterol esters in large HDL	*mmol/l*
	Free cholesterol in large HDL	*mmol/l*
	Triglycerides in large HDL	*mmol/l*
Medium HDL	*Concentration of medium HDL particles*	*mol/l*
	Total lipids in medium HDL	*mmol/l*
	Phospholipids in medium HDL	*mmol/l*
	Total cholesterol in medium HDL	*mmol/l*
	Cholesterol esters in medium HDL	*mmol/l*
	Free cholesterol in medium HDL	*mmol/l*
	Triglycerides in medium HDL	*mmol/l*
Small HDL	*Concentration of small HDL particles*	*mol/l*
	Total lipids in small HDL	*mmol/l*
	Phospholipids in small HDL	*mmol/l*
	Total cholesterol in small HDL	*mmol/l*
	Cholesterol esters in small HDL	*mmol/l*
	Free cholesterol in small HDL	*mmol/l*
	Triglycerides in small HDL	*mmol/l*

Lipoprotein particle size	*Mean diameter for VLDL particles*	*nm*
	Mean diameter for LDL particles	*nm*
	Mean diameter for HDL particles	*nm*
Cholesterol concentrations	*Total cholesterol*	*mmol/l*
	Total cholesterol in VLDL	*mmol/l*
	Remnant cholesterol (non-HDL and non-LDL cholesterol)	*mmol/l*
	Total cholesterol in LDL	*mmol/l*
	Total cholesterol in HDL	*mmol/l*
	Total cholesterol in HDL2	*mmol/l*
	Total cholesterol in HDL3	*mmol/l*
	Esterified cholesterol	*mmol/l*
	Free cholesterol	*mmol/l*
Glycerides and phospholipid concentrations (and one ratio)	*Total triglycerides*	*mmol/l*
	Triglycerides in VLDL	*mmol/l*
	Triglycerides in LDL	*mmol/l*
	Triglycerides in HDL	*mmol/l*
	Total phosphoglycerides	*mmol/l*
	Ratio of triglycerides to phosphoglycerides	
	Phosphatydilcholine and other cholines	*mmol/l*

	Sphingomyelins	*mmol/l*
	Total cholines	*mmol/l*
Apolipoprotein concentrations (and one ratio)	*Apolipoprotein A-1*	*g/l*
	Apolipoprotein B	*g/l*
Fatty acid concentrations	*Total fatty acids*	*mmol/l*
	Estimated degree of saturation	
	22:6, docosahexaenoic acid	*mmol/l*
	18:2 linoleic acid	*mmol/l*
	Omega-3 fatty acids	*mmol/l*
	Omega-6 fatty acids	*mmol/l*
	Polyunsaturated fatty acids	*mmol/l*
	Monounsaturated fatty acids; 16:1, 18:1	*mmol/l*
	Saturated fatty acids	*mmol/l*
Fatty acid ratios	*Ratio of 22:6, docosahexaenoic acid to total fatty acids*	%
	Ratio of 18:2 linoleic acid to total fatty acids	%
	Ratio of omega-3 fatty acids to total fatty acids	%
	Ratio of omega-6 fatty acids to total fatty acids	%
	Ratio of polyunsaturated fatty acids to total fatty acids	%

	Ratio of monounsaturated fatty acids to total fatty acids	*%*
	Ratio of saturated fatty acids to total fatty acids	*%*
Glycolysis related metabolite	*Glucose*	*mmol/l*
	Lactate	*mmol/l*
	Pyruvate	*mmol/l*
	Citrate	*mmol/l*
	Glycerol	*mmol/l*
Amino acid concentrations	*Alanine*	*mmol/l*
	Glutamine	*mmol/l*
	Glycine	*mmol/l*
	Histidine	*mmol/l*
branched	*Isoleucine*	*mmol/l*
branched	*Leucine*	*mmol/l*
branched	*Valine*	*mmol/l*
aromatic	*Phenylalanine*	*mmol/l*
aromatic	*Tyrosine*	*mmol/l*
Ketone body concentrations	*Acetate*	*mmol/l*
	Acetoacetate	*mmol/l*
	3-hydroxybutyrate	*mmol/l*

Fluid balance marker	*Albumin*	*mmol/l*
	Creatinine	*mmol/l*
Inflammation marker	*Glycoprotein acetyls, mainly a1-acid glycoprotein*	*mmol/l*

VLDL: very low density lipoprotein; LDL: low density lipoprotein; IDL: intermediate density lipoprotein; HDL: high density lipoprotein

Reference

1. Würtz, P. *et al.* Lipoprotein subclass profiling reveals pleiotropy in the genetic variants of lipid risk factors for coronary heart disease: a note on Mendelian randomization studies. *Journal of the American College of Cardiology* vol. 62 1906–1908 (2013).
